# Looking for an explanation for the excessive male mortality in England and Wales since the end of the 19th century

**DOI:** 10.1016/j.ssmph.2020.100584

**Published:** 2020-04-13

**Authors:** Valeria Maiolo, Alice M. Reid

**Affiliations:** a PhD Student at the Magna Græcia University of Catanzaro, Department of Legal, Historical, Economic and Social Science, Viale Europa, 88100, Catanzaro, Italy; b Cambridge Group for the History of Population and Social Structure, Department of Geography, Sir William Hardy Building, Downing Place, Cambridge, CB2 3EN, UK

**Keywords:** Decomposition-analysis, Age-cause-specific mortality, Excess male mortality, Life expectancy, Mortality

## Abstract

Several papers have primarily considered a female disadvantage in mortality as something to explain, considering a male disadvantage to be a “natural condition”. Even if, due to biological reasons, shorter life expectancy among males has been demonstrated, other factors need to be involved to explain firstly the increasing, and then the decreasing, of the male relative disadvantage over the past century.

The principal aim of this paper is to provide a clearer picture of the major age-class and cause-of-death contributions to male excess mortality in England and Wales from 1881 to 2011.

Results indicate a clear shift in contributions to the male disadvantage from differences occurring during the first year of life to those occurring in ageing people, and from tuberculosis, respiratory diseases, external causes and perinatal and congenital conditions to neoplasms and circulatory diseases. In contrast, the narrowing of the gap since 1981 seems to be most closely related to the decrease in the male disadvantage in respiratory diseases and to the simultaneous increasing in the female disadvantage in old-age diseases.

The most important novelty of this research relates to the method: instead of using *ratios* to investigate gender differences in health, we use decomposition methods.

## Highlights

• Excess male mortality increased from the 1880s until the 1970s, then declined.
• Until the 1930s infant mortality was a major part of higher male mortality.
• Cancer and circulatory disease dominated male excess mortality in the 20^th^ century.
• Female disadvantage from dementia and senility contributes to the declining sex gap in mortality.

## 1 Introduction

One of the great accomplishments of the twentieth century has been a rapid decline in mortality and the resulting gains in life expectancy (Elo & Drevenstedt, 2004). This improvement has not benefited both sexes equally, resulting in a widening of the sex difference in mortality. The female advantage in life expectancy in developed countries, which was only about 2–3 years around 1900, has increased to 8 years in recent decades (Horiuchi, 1999).

**Fig. 1:**
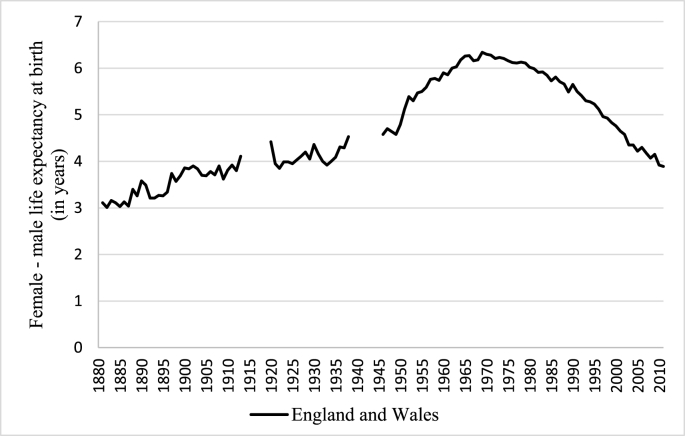
Sex differences in life expectancy at birth in England & Wales. Source: Authors’ elaboration on data from the Human Mortality Database. Note: Data covering the First and the Second World Wars were removed due to the excessive male mortality during those periods. 1919 data was removed because of the Spanish Flu, and this topic is not analysed in this paper.

In England and Wales, as in other European countries in the early nineteenth century, females enjoyed an overall longevity advantage. However, analysis of age-specific mortality rates shows they tended to die at higher rates than males at some ages when modern life tables show female advantage (McNay, Humphries, & Klasen, 2005). Moreover, males have experienced an intensification in their mortality disadvantage over time, which extended to all age groups during the 20^th^ century. Male excess mortality expanded during the long-term demographic/epidemiological transition in which infectious disease mortality was replaced by chronic disease mortality among adults (Beltrán-Sánchez, Finch, & Crimmins, 2015). Fig. 1 shows that female life expectancy in England and Wales exceeded that of males by about 3 years in 1881 and by more than 6 years between the 1960s and the 1970s. Since then, the gap has narrowed.

Increasing relative mortality for men is often described as a male epidemic (Lawlor, Ebrahim, & Davey Smith, 2001). However, given today's female mortality advantage across the whole lifespan, much of the literature focuses on the excess female mortality shown in some age classes in the past, as being the experience that requires explanation. In contrast, this paper wants to focus on the excess in male mortality and on the factors involved in that.

Hinde (2011 p. 13) argues that the terms “excess female mortality” and “excess male mortality” are “ambiguous” because they “imply that the standard is equality between the sexes”. Actually, the norm is not equality: several studies have demonstrated a “natural” female advantage in life expectancy of about 1-2 years (Pressat, 1973; Trovato & Lalu, 1996). However, the aim of this paper is not to focus only on non-natural factors, but on the whole range of factors determining the sex gap in mortality, and, in particular, on higher male mortality. In these terms, it is possible to assume that, without the contribution of natural and not natural factors, the sex gap in longevity would not exist. Following this assumption, the implication of sex equality in life expectancy becomes true and the use of the term excess female mortality simply indicates female mortality higher than male mortality, and excess male mortality indicates male mortality higher than female*.*

As mentioned above, the gap between male and female mortality can be explained by a combination of biological, genetic and behavioural factors. The biological factors are largely beyond human control and are sometimes also called “inherited risks” (Luy, 2016). Supporters of biological explanations highlight that higher male mortality rates are found among infants and children, ages at which differences in behaviour play a modest role (Gjonça, Tomassini, Toson, & Smallwood, 2005). In all developed countries, there is 25 percent excess male mortality up to 5 years, and this occurs even in countries with various ethnicities and medical systems (Mage & Donner, 2015). Several hypotheses have been proposed regarding the role of sex associated genetic and endocrine differences in the determination of neonatal mortality or morbidity (Zhao, Zou, Lei, & Zhang, 2017). For example, Naeye, Burt, Wright, Blanc, and Tatter (1971) proposed that male excess mortality in this age group was linked to damage to the X-chromosome. They analysed neonatal mortality in the first 72 h of life and concluded that “the biological differences must originate in the genetic differences between the sexes, and those genetic differences are a consequence of disparity in number of the X chromosomes”. Men possess only one X chromosome, while women possess two, so if one X chromosome is damaged, among women the second one can compensate.

There are biological differences which can impact on sex differences in health in adulthood as well. For example, it has been shown that women’s sex hormones reduce the risk of ischemic heart disease, while men’s hormones tend to increase that risk (Maas & Appelman, 2010). Higher testosterone also contributes to the undertaking of hazardous behaviour, resulting in a higher rate of accidental and violent deaths among men (Gjonça et al., 2005). However, many studies have found that the natural female survival advantage is responsible for only a minor fraction of the increased differences in life expectancy between men and women in developed countries (Luy, 2016). Studies using data for Mormons, Seventh-day Adventists, Old Order Amish etc. demonstrate significantly lower male excess mortality (Luy, 2016) in communities where female and male lifestyles are more similar.

For this reason, the role of other factors, the non-biological ones, seems to be unquestionable. Non-biological factors include behavioural, cultural and environmental factors, also identified as factors directly or indirectly influenced by human action, and often called “acquired risks” (Luy, 2016). The importance of these risks has been influenced by differences in gender roles, which have led to greater exposure of men than women to health risks, such as smoking, drinking, injuries, and violence (Pampel, 2003; Waldron, 1995). Indeed, a gender equalisation hypothesis has been suggested, which proposes that as women’s roles become more similar to those of men, a narrowing of the sex difference in mortality would be observed (Pampel, 2003). As Hinde (2011) argues, differentials in mortality are important indicators of the ways in which male and female roles and behaviour in a population differ, and of the relative status of males and females within a society.

In the discussion so far we have used ‘sex’ to refer to biologically-related effects and ‘gender’ to refer to the effects of behavioural and lifestyle factors, however the distinction is not always clear cut (for example the contribution of testosterone to higher risk-taking behaviour among men blurs the distinction). Moreover when reporting mortality and causes of death it is not always obvious whether differences between men and women are due to factors related to sex or to gender, and therefore in the rest of this paper our default word is ‘sex’.

Identification of the factors involved in sex disparities in mortality is the first step to understanding the determinants of the time-trend in the sex gap. However, in order to identify these factors, it is important to calculate the contribution of age-classes and of leading causes of death to the total sex gap.

Nowadays, the major source of male excess mortality in industrialized countries is cardiovascular diseases (CVD). The contribution of this cause-of-death group to the sex disparity in mortality has constantly increased, especially since the middle of the twentieth century. Many hypotheses have been advanced in order to explain this major form of male mortality. For example, changes in smoking and diet and other behavioural or lifestyle factors may have affected men more than women (Beltrán-Sánchez et al., 2015). Biological explanations have also been suggested (Maas & Appelman, 2010). However, the causes of excess CVD in males remain unclear: in Nikiforov and Mamaev’s (1998) opinion, “efforts to pinpoint the causes of the disparity have been hampered by a lack of understanding of the basic historical trends in sex differences in CVD” (p. 1348). In our opinion, this statement could and should also be applied to the entire nosological framework.

For this reason, the aim of this paper is to study the contributions of differences in life expectancy at specific age classes, and from specific causes of death, to the total male disadvantage, and how these have changed over time.

This paper aims to add to the literature on sex differences in mortality by examining the age and the cause-specific contributions to sex differences in mortality in England and Wales between 1881 and 2011. Although there have been several previous investigations of sex differences in life expectancy in England and Wales, those tend to focus on female mortality or they do not provide a definite explanation of excessive male mortality (e.g. Hinde, 2011; Martin, 1951; McNay et al., 2005). Other studies of England and Wales use mortality ratios and generally do not examine a very long-time frame (Gjonça et al., 2005; Reid, Garrett, Dibben, & Williamson, 2016 b; Trovato & Heyen, 2006). This paper considers not only a longer time frame, but a distinctive methodology. To achieve the paper’s aims, sex disparities are studied using decomposition methods instead of the sex-ratio in mortality rates or life expectancy.

## 2 Data and methods

### 2.1 Data

Data were collected for the census years 1881, 1891, 1901, 1911, 1921, 1931, 1951, 1961, 1971, 1981, 1991, 2001, 2011. Census years were chosen to enable precise conjunction with populations at risk, which are the most accurate for those years. There was no census taken in 1941 due to the Second World War, so 1939 is used instead, when a national register of population was taken for rationing and conscription purposes.

To decompose the total sex gap by age, data about life expectancy and about the number of people who survive to age *x* years must be collected. These data for England and Wales were extracted, already computed, from the period-life table (England and Wales, Total Population, Period Life tables,1x1, Males and Females) provided by the Human Mortality Database (hereafter cited as HMD). All the data used were collected for both sexes, for the age groups 0–11 months, 1–14 years, 15–29, 30–44, 45–59, 60 onwards. The size of each age class is fifteen years, except for the first, the second and the last classes. The size of the first age class is only one year due to its very high impact during the early periods under examination and the fact that the cause of death profile for infants is quite different to that of older children. As a consequence of the size of the first age class, the size of the second is only 14 years. The last age class, from 60 years old onwards, is an open-ended age group. On average, it is no larger than the others if we consider that the data for England & Wales show that female life expectancy at birth was under 75 years old until the 1970s, and that of males exceeded 75 only in 1999 (HMD). In 2011 life expectancy was 83 for females and 79 for males (HMD).

To decompose the total sex gap by cause of death, it is necessary to collect the numbers of deaths by sex, age, and cause of death, and the data about the population composition. The total number of deaths - always by sex and age - are also required.

The population data by single year of age and sex were obtained from the Human Mortality Database. The cause of death data were not available for the six broad age groups that we used to decompose the sex gap by age. The original nineteenth-century tables produced numbers of deaths from each cause for nine, slightly different age classes: 0–11 months, 1–14 years, 15–24, 25–34, 35–44, 45–54, 55–64, 65–74, 75 onwards. Later data provided more detailed age groups, but smaller units were combined to form the nineteenth century age groups in order to permit comparisons over time. To allow calculation of the mortality contributions by age group and cause of death, the population data and the contributions by age were arranged into the same age groups used by the original cause of death tables.

The numbers of deaths in England and Wales from different causes, based on the causes of death recorded on death certificates, were classified and tabulated according to age group and sex and published by the Registrar-General of England and Wales. Here we have used machine readable databases of these tables made available by different bodies. The data for 1881 and 1891 were compiled by Romola Davenport (Davenport, 2007). The rest of the data were downloaded from the Office for National Statistics: for 1901 to 1999 the source was “The 20th Century Mortality Files, 1901–2000 release”; 2001 data were taken from “21st Century Mortality dataset, England & Wales 2001–16”; and 2011 data were from “Deaths registered in England and Wales, 2011 - Table 5” (Office for National Statistics, nd a, nd b, and 2017).

Over time there was a dramatic increase in the number of different causes or causal groups reported: in 1881 and 1891 only 173 distinct causes were reported, but in 2011 numbers of deaths were provided for over 3000 individual causes. Comparing causes of death over time can be very problematic, at least partly because of the frequent changes in the nosologies employed in the Annual Reports. Nosologies can be affected by changes in nomenclature because of the transfer between broad categories as new theories regarding causal agents led to a re-grouping (Reid et al., 2016a, Reid et al., 2016b). In addition, in the nineteenth century many causes were vague or poorly defined, due to a combination of the lack of routine autopsy, rudimentary knowledge of the symptoms of many conditions, and a lack of medical attendance during the final illness (Reid et al., 2016 b). Complications with cause of death data did not disappear with the introduction of the various revisions of the International Classification of Diseases (ICD). The ICD can be defined as a system of categories to which morbid entities are assigned according to well specified criteria (WHO n.d.). The purpose of the ICD is to permit the systematic analysis, interpretation and comparison of mortality and morbidity data collected in different countries or areas and at different times. However, since its first adoption, the ICD has been changed several times. Every revision can affect time trends in cause-of-death statistics (Gjertsen, Bruzzone, Vollrath, Pace, & Ekeberg, 2010).

Perhaps the largest problem in comparing causes of death over time is the fact that deaths attributed to unknown, poorly defined or symptomatic causes reduced dramatically over time: as medical attention during illness increased, medical knowledge improved, and the frequency of autopsy rose, more deaths were assigned to a cause, and these causes were increasingly accurate. This makes creating a consistent cause of death grouping for use over time particularly difficult. Many studies take the approach of aggregating up individual causes where there is no straightforward transfer of a cause from one group to another: this can be useful over relatively short time frames when there are few nosological changes, but over longer time scales it tends to end up with large proportions of deaths in ‘nosologically not meaningful’ categories (Wolleswinkel-Van den Bosch, Van Poppel, & Mackenbach, 1996) and does not solve the problem of the ill-defined causes.

Other studies redistribute the ‘unknown’ and ‘ill-defined’ causes of death into other categories (Meslé & Vallin, 1996). However, this is also problematic as it involves making assumptions about the proportions of such deaths which should be redistributed to each of the other categories (Janssen & Kunst, 2004, p. 911; Reid, Garrett, Dibben, & Williamson, 2015, pp. 323–324). This is unsatisfactory because it is very likely the balance between different ‘real’ causes among the ill-defined changed over time. In the mid nineteenth century, for example, infant deaths formed a large proportion of all deaths, and the causes of a great many infant deaths were also, according to death certificates, ‘unknown’. Deaths among the elderly were very likely to be attributed to the ill-defined cause of ‘old age’. Mortality declines started with infectious diseases among younger adults and children, ages with relatively few unknown or ill-defined deaths, followed by declines in infant mortality. Mortality improvements in old age had to wait longer, and thus the composition of the ‘ill-defined’ category will have changed considerably over the period from 1881 to 2011.

Our approach to the formation of a classification over time starts with the fifteen groups and their membership used by Reid et al., 2015, Reid et al., 2016a, Reid et al., 2016b who began by allocating causes to an ICD10 chapter, and then re-allocated causes between chapters in order to achieve consistency within categories over time (for more details see Reid et al., 2015, p. 324). As part of this process several symptomatic causes were combined with more clearly diagnosed causes occurring in the same area of the body (for example ‘pleurisy’ was placed with ‘diseases of the respiratory system’). Some chapters with small numbers were merged, and some groups of causes which were particularly important in the nineteenth century, either in numerical terms or as foci of public health and medical concern, were retained as groups, specifically diarrhoea-like diseases, tuberculosis, and old age. These groups were so significant at the beginning of the period in terms of number of deaths that they deserve to be considered separately. They were as significant as the circulatory group and larger than that of neoplasms (see Appendix B sheet “Total deaths”). Declines in diarrhoea and tuberculosis in particular have been singled out as major causes of mortality declines in particular age groups, and tuberculosis has also been implicated in differences in mortality between the different sexes (Hinde, 2015; McKeown, 1976; Reid & Garrett, 2018; Szreter, 1988).

We used Reid et al.'s classification of causes up to the year 1939, and we extended their classification into subsequent years, with very minor changes to ensure consistency over time. Finally, we combined the ‘other causes’ and ‘childbirth’ categories used by Reid et al. in order allow sex comparisons between all groups of causes.

Our resulting classification into fourteen groups therefore strikes a balance between retaining internal consistency within groups over time, and providing distinct groups which are particularly relevant at various periods and which may respond to influences related to the sex-gap in mortality. This does not mean, of course, that the issues of differential diagnosis over time have been solved. The ‘ill-defined’ category is still larger at the start of the period than the end, and there are bound to have been deaths which would have been classed in different causal groups at different times, an issue which will particularly affect the ‘old age’ category. We comment on these issues as we interpret our results.

Appendix A shows our fourteen groups (Genitourinary, Nervous system, Digestive, Perinatal & congenital, Old age, Tuberculosis, Circulatory, Neoplasms, Infectious, Diarrhoea, Respiratory, External causes, Other + Childbirth, Ill-defined) and the main ways in which they differ from ICD10 chapters. Because of the very modest role of the Diarrhoea group as a cause of death in 2001 and in 2011, for these years this group has been redistributed between Infectious diseases and Digestive diseases, depending on the specific cause. Appendix B provides detailed lists of the original causes of death and how they were classified.

### 2.2 Methods

As Hinde (2011) argues, “one obvious way to begin to address the relationship between social, economic and cultural factors and mortality differentials is to look at age-specific mortality rates” (p. 14). The most popular measures of sex differences in life chances are the female/male *ratio* of the age-specific probability of dying (*_n_q_x_*) and the closely related female/male *ratio* of age-specific mortality rates (*_n_*m*_x_*) (McNay et al., 2005). In this paper, decomposition methods instead of *ratios* are preferred. The *ratio* suffers from the problem that at ages where mortality is low, quite extreme *ratios* may be associated with very small differences between the sexes in the numbers of deaths, and hence with a very small impact on the overall sex differential in mortality (Hinde, 2011).

In this paper, the total sex gap is firstly decomposed by age using the United Nations' (1982) method, and secondly by cause of death using the method developed by Preston, Heuveline, and Guillot (2001, p. 291).

Several methods have been proposed for the decomposition of differentials in life expectancy at birth. Ponnapalli (2005), after comparing different decomposition methods, shows that the results of different methods are similar whenever appropriate formulae are applied to the same set of data. In this paper, the United Nations’ method is used because of its simplicity, intuitiveness and comprehensibility.

If, for a given population, the expectations of life for males and females at the age *x* years are denoted by the symbols e_*x*_^m^ and e_x_^f^ respectively, and the number of people who survive to age *x* years by the symbols *l*_*x*_^*m*^ and *l*_*x*_^*f*^ respectively, then the contribution of age-group *x* to *x + n* years, _*n*_Δ_*(x; x+n)*_, to the overall sex differential in the expectation of life at birth is given by the formula:

1. _*n*_Δ_*(x; x+n)*_ = [0.5 × (*e*_*x*_^*f*^ − *e*_*x*_^*m*^) × (*l*_*x*_^*f*^ *+* *l*_*x*_^*m*^)] − [(0.5 × (*e*_*x+n*_^*f*^ − *e*_*x+n*_^*m*^) × (*l*_*x+n*_^*f*^ *+* *l*_*x+n*_^*m*^)]

For the open-ended age group, the formula is:

2. _*n*_Δ_*(x)*_ = [0.5 × (*e*_*x*_^*f*^ − *e*_*x*_^*m*^) × (*l*_*x*_^*f*^ + *l*_*x*_^*m*^)]

The results of these formulae (_*n*_Δ_*(x; x+n)*_ and _*n*_Δ_*(x)*_) indicate the contribution in years of the mortality differential between ages *x* and *x* *+* *n* years to the total difference in life expectancy at birth (*e*_*0*_^*f*^ − *e*_*0*_^*m*^). Negative numbers mean that life expectancy is higher, i.e. mortality is lower, for males than females in that age group, namely that females have a disadvantage in term of health. Positive numbers mean a male disadvantage. In this paper, the difference between men’s and women’s life expectancies at birth was decomposed by the contributions of six age groups: <1-year-old, 1–14 years old, 15–29 years old, 30–44 years old, 45–59 years old -using formula 1-; 60 onwards -using formula 2-.

Using formulae 1 and 2, the results are the contribution of every age class to the total sex difference in life expectancy at birth. As stated above, when male life expectancy is higher than female, the results are negative numbers and vice-versa.

The second step is to estimate the contribution of differences in cause-specific death rates. Using Preston et al.’s method, the specific contribution of differences in mortality rates from cause *i* between ages *x* and *x* *+* *n*, _*n*_Δ_(*x; x+n)*_^*i*^, can be estimated by multiplying the proportion of deaths from cause *i* between ages *x* and *x + n* for female, *R*_(*x; x+n)*_^*i*^*^(f)^*, by all-cause mortality rate between ages *x* and *x* *+* *n* for females, *m*_(*x; x+n)*_*^(f)^*, minus the same for males; the whole divided by all-cause mortality rate between ages *x* and *x* *+* *n* for females minus that for males, *m*_(*x; x+n)*_*^(f)^* − *m*_(*x; x+n)*_*^(m)^,* and the result multiplied by the contribution of age-group *x* to *x* *+* *n* years to the overall sex differential in the expectation of life at birth, _*n*_Δ_*(x; x+n)*_.

In summary, we have the following equation:

3. ${}_{n}\Delta_{\left(x;x+n\right)}^{i}\;={\;}_{\;n}\Delta_{\left(x;x+n\right)}\;\times \;\frac{{R_{\left(x;x+n\right)}}^{{{}^{i}}^{\left(f\right)}}\;\times \;{m_{\left(x;x+n\right)}}^{\left(f\right)}\;-\;{{R_{\left(x;x+n\right)}}^{i}}^{\left(m\right)}\;\times \;{m_{\left(x;x+n\right)}}^{\left(m\right)}\;}{{m_{\left(x;x+n\right)}}^{\left(f\right)}\;-\;{m_{\left(x;x+n\right)}}^{\left(m\right)}}$

Given that:

*R*_*(x; x+n)*_^*i*^*^(f)^ =* deaths from cause *i* between ages *x* and *x + n* in the female population, divided by deaths from all causes between ages *x* and *x + n* in the female population;
*R*_*(x; x+n)*_^*i*^*^(m)^ =* deaths from cause *i* between ages *x* and *x + n* in the male population, divided by deaths from all causes between ages *x* and *x + n* in the male population;
*m*_*(x; x+n)*_*^(f)^ =* deaths from all causes between ages *x* and *x + n* in the female population, divided by the female population between ages *x* and *x + n;*
*m*_*(x; x+n)*_*^(m)^ =* deaths from all causes between ages *x* and *x + n* in the male population, divided by the male population between ages *x* and *x + n.*

In Formula 3, _*n*_Δ_(*x; x+n)*_^*i*^ represents the numbers of years of life gained by females by eliminating cause *i* minus the number of years of life gained by males by eliminating cause *i*, which is simply the sex differential in the number of years lost to a particular cause of death.

The age-and-cause contribution is provided in the paper only for selected years, namely for 1881, 1951, 1971, 2011. We decided to present these years as they represent different points of the gap: 1881 displays the situation at the beginning of the period, 1951 represents the period before the peak of the gap, 1971 represents the peak, and 2011 represents the most recent year of the analysis. The results for the other decades are available in Appendix C.

The cause contribution, however, is provided for every decade of the whole period and represents the sum of the cause-contributions from all age-groups.

Calculations were carried out using Microsoft Office Excel 2010 (Microsoft Corporation, 2010).

## 3 Results

**Fig. 2:**
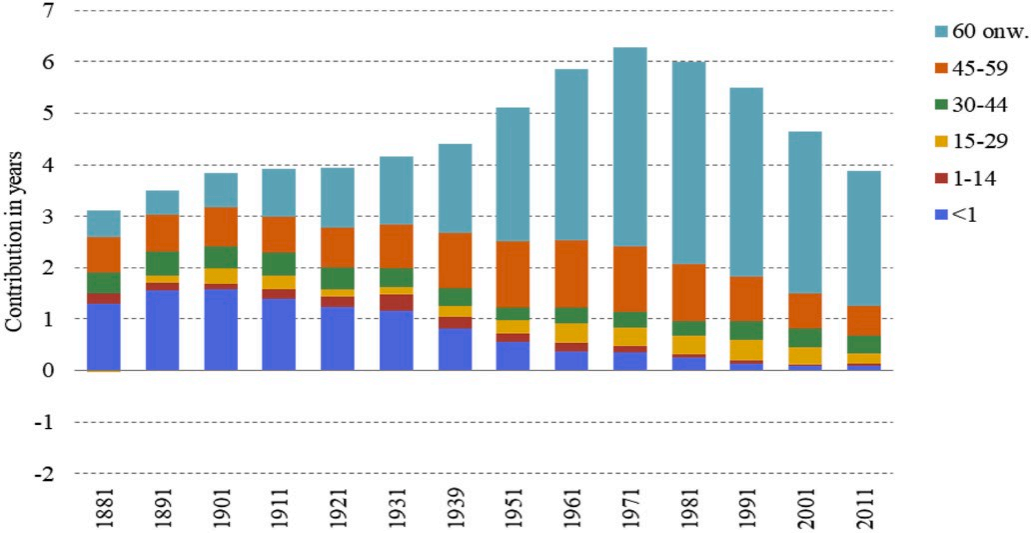
Contribution in years of sex-differences in life expectancy by age to the total sex gap. Note: Positive contributions indicate higher male mortality. Source: Authors’ elaboration on data from the Human Mortality Database. (For interpretation of the references to colour in this figure legend, the reader is referred to the Web version of this article.)

Fig. 2 shows the results of the decomposition of the total sex gap by age. It displays higher male mortality during the first year of life across the whole period under consideration. This had a profound effect on male disadvantage in life expectancy at the beginning of the period. For example, in 1881 the male disadvantage in infant mortality contributed 1.30 years to the total difference in life expectancy of 3.11 years. Since the beginning of the twentieth century its relative contribution has consistently decreased over the time, in line with the reduction in infant mortality.

Looking at age classes from 1-44 years old, their contributions, even if interesting, are small and irregular and they do not help to elucidate the general picture. In contrast, the contributions of the age classes from 45 onwards are the most important in explaining the evolution of the male disadvantage across the last century. In particular, the contribution of the 45-59 year-old age class to the total male disadvantage shows firstly a rise and then a fall, with a peak between 1939 and 1961. The absolute contribution of the age class from age 60 onwards also exhibited a rise and then a fall, peaking between 1970 and 1980. However, in relative terms the contribution of this age group increased constantly over time, explaining more than the 60% of the higher male mortality at every year since 1971.

**Fig. 3:**
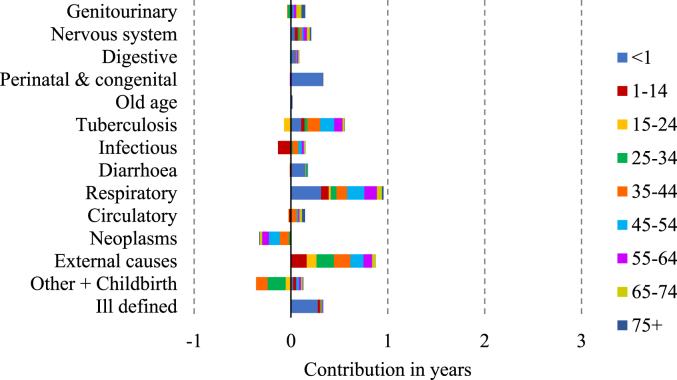
Age-cause-contribution in years to the total sex gap in 1881. Note: Positive contributions indicate higher male mortality. Source: Authors’ elaboration on data from several sources. (For interpretation of the references to colour in this figure legend, the reader is referred to the Web version of this article.)

**Fig. 4:**
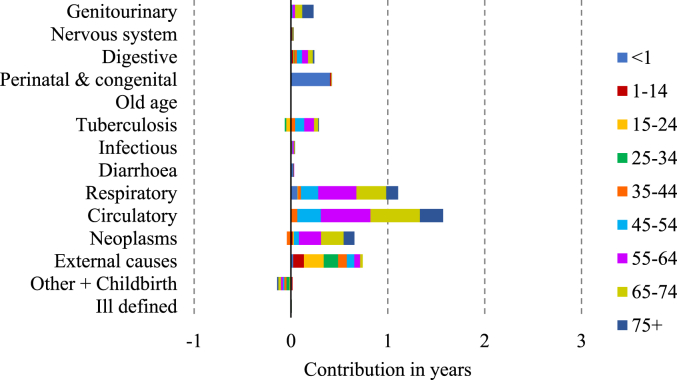
Age-cause-contribution in years to the total sex gap in 1951. Note: Positive contributions indicate higher male mortality. Source: Authors’ elaboration on data from several sources. (For interpretation of the references to colour in this figure legend, the reader is referred to the Web version of this article.)

**Fig. 5:**
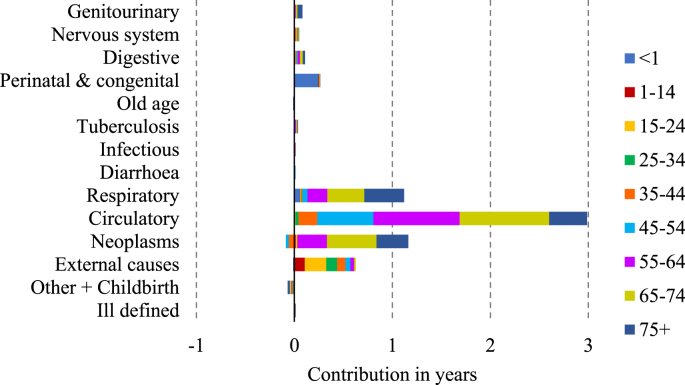
Age-cause-contribution in years to the total sex gap in 1971. Note: Positive contributions indicate higher male mortality. Source: Authors’ elaboration on data from several sources. (For interpretation of the references to colour in this figure legend, the reader is referred to the Web version of this article.)

**Fig. 6:**
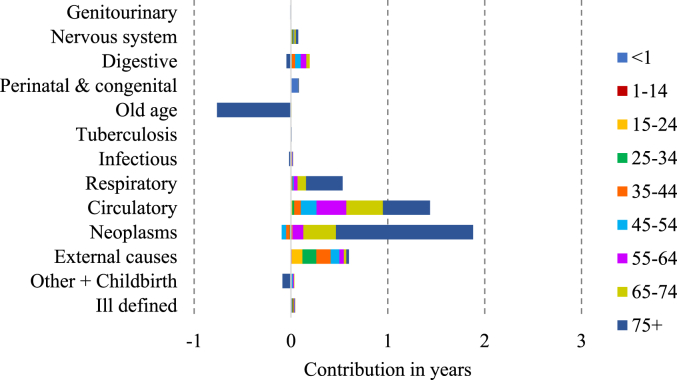
Age-cause-contribution in years to the total sex gap in 2011. Note: Positive contributions indicate higher male mortality. Source: Authors’ elaboration on data from several sources. (For interpretation of the references to colour in this figure legend, the reader is referred to the Web version of this article.)

**Fig. 7:**
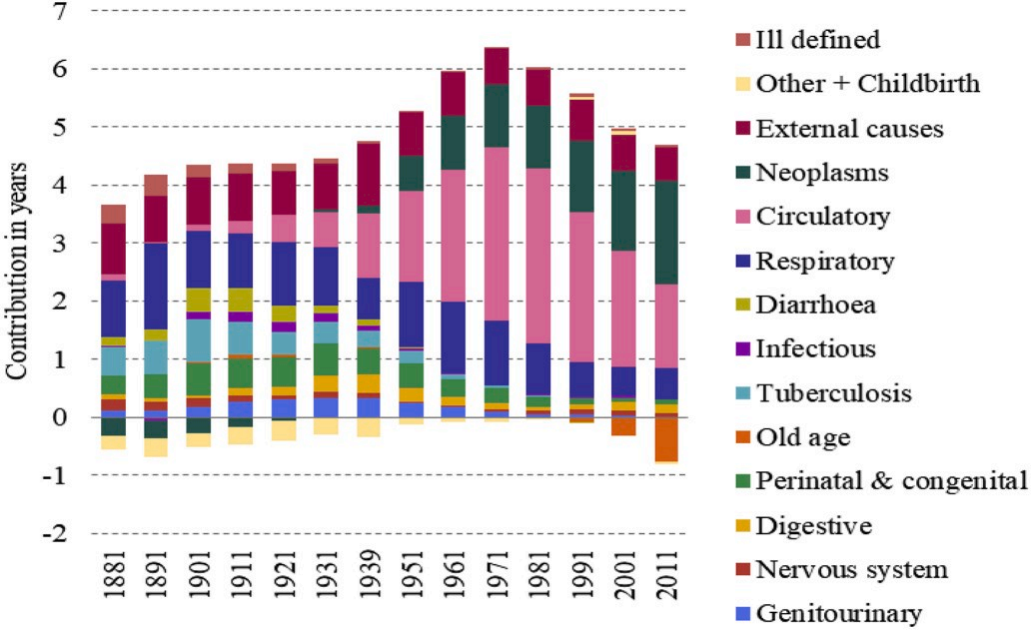
Cause-contribution in years to the total sex gap from 1881 to 2011. Note: Positive contributions indicate higher male mortality. Source: Authors’ elaboration on data from several sources. (For interpretation of the references to colour in this figure legend, the reader is referred to the Web version of this article.)

Fig. 3, Fig. 4, Fig. 5, Fig. 6 display the age-cause contribution to the total sex gap in life expectancy at birth in 1881, 1951, 1971, 2011 respectively. Fig. 7 shows the total cause-contribution to the total sex gap for the whole period, from 1881 to 2011.

At the beginning of the period females seem to have suffered a disadvantage in mortality from neoplasms and from the other-cause group (Fig. 7). The disadvantage from neoplasms is concentrated at ages from 35 to 64 years old, and that from other diseases and childbirth involves the age class from 25 to 34 (Fig. 3). This female disadvantage from neoplasms could be related to the limited diagnostic tools of the period: the proportion of diagnoses of death from breast cancer which were accurately diagnosed was probably higher than that for lung cancer or for the common male cancers, such as prostate cancer, because the signs of breast cancer were easier to identify. However, the female disadvantage from the other-cause group can be considered to be more realistic and related to female mortality due to childbearing and delivery.

Another observation about female mortality is that the aggregate pictures shown in Fig. 2, Fig. 7 conceal a tendency for women and girls to have died at higher rates than men and boys in certain age- and cause-groups. For example, a female disadvantage is visible for infectious diseases from 1-14 years old in 1881 displayed in Fig. 3, while the overall value for infectious diseases in this period shows a male disadvantage (Fig. 7).

Focusing on the male disadvantage shown at the beginning of the period, higher relative male mortality was due to mortality from respiratory diseases, external causes, perinatal and congenital causes, and from tuberculosis.

Tuberculosis has often been thought to be a predominantly female disease. However, this idea is questionable. Hinde (2015) studying the spread of phthisis, or pulmonary tuberculosis, in England and Wales in 1861 and 1871, found that the sex ratio of deaths from phthisis varied greatly from place to place and, in some places where phthisis was prevalent, men rather than young women were at the greatest risk of death. Reid and Garrett (2018) show that tuberculosis mortality among young adults in Scotland was higher for men in rural areas and for women in those urban areas where textile work was common. They argue that all textile workers were particularly vulnerable to the disease but female tuberculosis mortality was higher in textile towns because there were many more female textile workers than male. Martin (1951) found that in England and Wales in the middle of the twentieth century, the sex ratios in mortality from tuberculosis and from diseases of the respiratory system, which were at a maximum at ages 45–55, showed a male death rate three times that of females. As Dinges and Weigl (2016) argued “The incidence of tuberculosis varied depending on the time of life: male excess mortality due to tuberculosis occurred mostly in infancy, while in women it occurred mostly below the age of twenty. Male excess mortality prevailed consistently in adulthood” (p. 199). This is in line with what Fig. 3, Fig. 4 display: the contribution of this cause of death by single age classes shows that excess female mortality from tuberculosis is registered for several age classes (particularly from 15 to 24 years old), but it was out-weighed by the relative higher male mortality in other age classes.

Differences in mortality from respiratory diseases and from perinatal deaths contributed significantly to the male excess mortality during the first year of life, while the contribution of external causes was spread over several age-classes (Fig. 3).

Going forward in time, the role of tuberculosis and childbirth-related death was exhausted and replaced by circulatory diseases and neoplasms, from which the excess female mortality turned into a male excess in the 1930s (Fig. 7). The relative contributions of respiratory diseases and external causes to male mortality, although still important, have been decreasing over time (Fig. 7).

The peak in the male disadvantage in life expectancy is very likely explained by sex-specific mortality from circulatory diseases and neoplasms, which characterise the male disadvantage, in particular for men over 45 (Fig. 4, Fig. 5). On the other hand, the narrowing of the gap since 1981 seems to be most closely related to the decrease in the male disadvantage in respiratory diseases and to the simultaneous increasing in the female disadvantage in old-age diseases (Fig. 7). This female increment seems to be particularly related to several type of dementia and senility which are included in this cause-of-death group.

Finally, the contribution of differences in mortality from circulatory diseases has also decreased but has been compensated by the increment of sex differences in mortality from neoplasms.

## 4 Discussion

Fig. 2 shows the contribution of sex disparities in life expectancy by age to the total sex gap. What is surprising, looking at the first part of the graph, is the very high contribution of the male disadvantage from a single year of life: the first. It shows no signs of diminishing before the middle of the twentieth century.

On the average baby boys have the same environment as baby girls; therefore, circumstances and behaviors that may have a differential effect on male and female adult mortality should play no part in the difference between the sexes in infancy (Martin, 1951). For this reason, the male disadvantage during the first year of life is very likely to be a consequence of biological factors. As mentioned above, males are more prone to prematurity, respiratory problems and to foetal distress (Mage & Donner, 2015; Naeye, 1971). The decline of the contribution of this age class, shown in Fig. 2, has to be considered as a result of the decline in infant mortality which occurred as a consequence of the advances in obstetric and neonatal care and of the decrease in infectious diseases. Both of these are likely to have benefitted males more than females (Reid et al., 2016 b), partly because the number of deaths at this age class was higher among males and also because obstetric advances reduced the negative effect of difficult deliveries, to which baby boys were particularly vulnerable. With the decrease in infant mortality, the major contribution to males’ disadvantage has shifted from the impact of that occurring during the first year of life, to that occurring in ageing people. This shift is very likely a consequence of the sex differences in the mortality pattern experienced in several European countries due to the epidemiological transition. The epidemiological transition is characterised by a shift in the age pattern of mortality effected by larger declines in infant and child mortality than at older ages, and this can explain the decrease in the importance of the first year of life. The epidemiological transition theory also depicts a change in the balance of causes of disease from infectious to so-called “man-made diseases”, but it does not provide any explanation of why the relative increase in mortality from the latter has affected males more than females.

One explanation is that as mortality fell, the large male mortality disadvantage which emerged may have been due to the elimination of gender-neutral causes of death such as infections, and female-specific causes of death such as maternal mortality, leaving a larger role for circulatory diseases and neoplasms, which particularly characterise the male disadvantage since the middle of the twentieth century. But, if the male disadvantage during the first year of life seems to be clearly related to biological factors, what factors are responsible for the male disadvantage in adulthood from circulatory diseases and neoplasms?

As stated in the introduction, the literature offers a list of hypotheses which can be summarised under the following groups: biological differences, social-role differences, cigarette-smoking differences. Explanations of biological differences in adulthood, as mentioned above, focus particularly on differences in hormone prevalence. This hypothesis, although not implausible, is not consistent with the trend of the sex-differences in mortality over time. In addition, variation in the sex-gap in longevity between countries indicates that the difference in mortality between the sexes is not purely biological and that social factors must be taken into account.

Social-role differences emphasise the fact that males tend to be employed in more dangerous, harmful, and difficult occupations. However, even if excess occupational risks in males are important in determining the size of the sex differences in mortality, the actual assessment of their influence is difficult (Martin, 1951). It is likely that occupational and social risk-taking behaviour are a long-standing feature of societies and did not only appear in the 1920s. Moreover, the second part of the 20th century is characterized by increasing attention to the prevention of occupational hazards (Nikiforov & Mamaev, 1998). Over the last century, hours of work have been curtailed, and many of the gross industrial risks have been minimized. It would be expected, therefore, that any improvement in conditions of work would affect males more than females, yet the female death rate has continued to decline faster than the male (Martin, 1951), and it is therefore unlikely that the mid-twentieth century increase in the male disadvantage was linked to changes in work conditions. However, as a consequence of industrialization, there was a shift from the prevalence of primary-sector employment to the secondary and tertiary sectors. While the sex-composition of the labour force in agriculture was quite even, that in the secondary sector was not, with continued concentration of men’s occupations in more dangerous work, which remained more dangerous even after the improvement of conditions. More investigation about the link with labour activity is needed; all it is possible to say now is that the improvement of work-conditions could partly explain the decrease of the contribution of deaths from external causes - in which deaths from accidents are included-, and to which males have been more prone. It is also important to remember that the relationship between work-risks and health is not limited to accidents, but also includes the increase of risk of mortality from other causes like neoplasms or respiratory diseases. This is the case, for example, with asbestosis and of pneumoconiosis which are occupation-specific illnesses suffered by people who worked with particular materials.

Focusing on the final hypothesis, smoking is very likely to have played an important role in explaining the trend of the sex differences in life expectancy since the middle of the twentieth century in England & Wales and in other developed countries as well. Clear differences between males and females have been observed in smoking, in terms of both the propensity to smoke and trends over time.

**Fig. 8:**
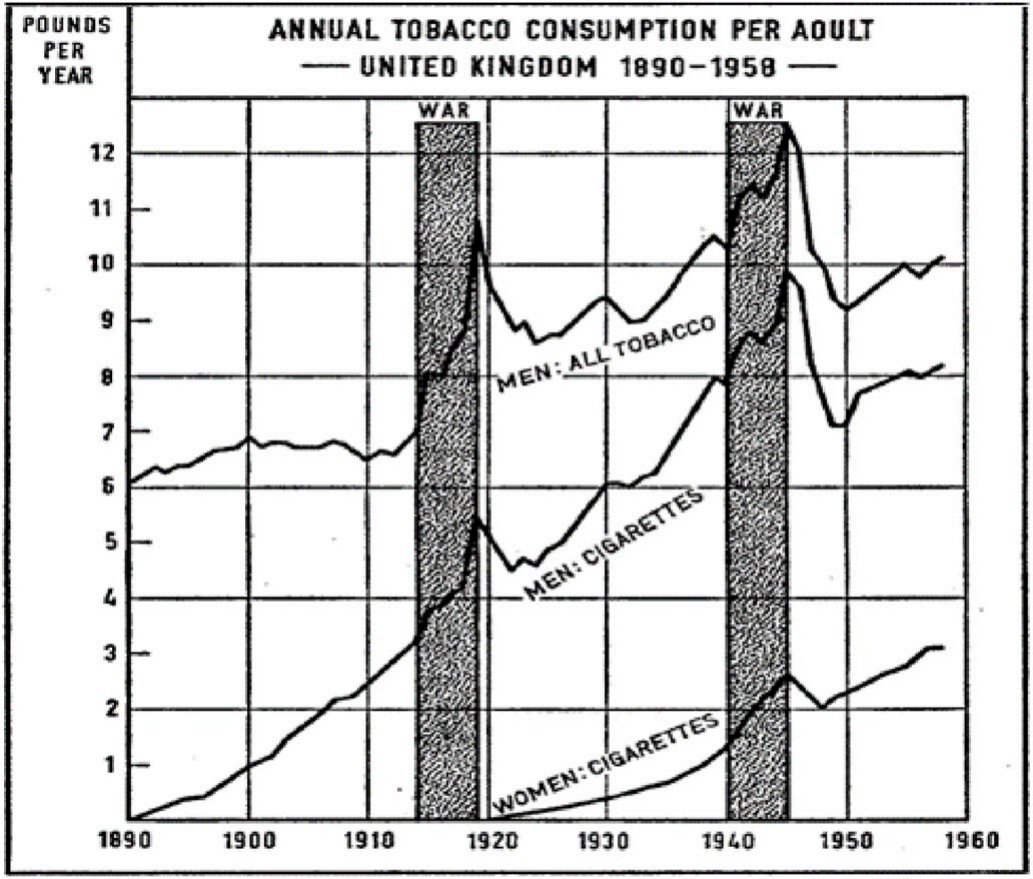
Annual tobacco consumption per adult. Source: Royal College of Physicians of London (1962, p. 81), “Smoking and Health” (p. 14).

As Fig. 8 shows, men’s consumption of tobacco, mainly in the form of pipe smoking, had been common during the late nineteenth century, but cigarette consumption was negligible until the final years of the century and really picked up pace during the First World War, with a further peak during the Second World War. Women’s consumption of cigarettes did not start until the 1920s, and only began to approach that of men after the Second World War; however, the number of cigarettes smoked for women remained lower than that for men (Royal College of Physicians of London, 1962). These differences reflect strong differences in the propensity to smoke by birth cohort: over 80 per cent of men born between 1897-1901 and 1922–1926 smoked at some point of their lives, but men born later were progressively less likely to have smoked (Kemm, 2001). In contrast few women born in the early twentieth century ever smoked, and the peak of cigarette smoking in women occurred in cohorts born during the 1920s (Kemm, 2001). People tend to suffer from smoking-related diseases when they reach middle and old age, so from the 1950s–1970s, when the men who had started smoking during the First World War were of an age to develop such diseases, women of the same age were not so vulnerable because they were predominantly non-smokers. This is particularly visible in Fig. 4, Fig. 5, shown by the male disadvantage due to their higher mortality from respiratory and circulatory diseases and neoplasms in the age classes from 55 to 74 years old. The different prevalence of cigarette-smoking between the sexes is likely to be related to the different sex-specific mortality from several neoplasms, in particular from lung cancer. Cigarette smoking also increases CVD mortality. Male smokers between the ages of 45 and 64 have a 90% higher CVD death rate than male non-smokers (Nikiforov & Mamaev, 1998).

The contribution of cigarette consumption to the trend of neoplasms and circulatory diseases and the peak of the sex gap is unquestionable, but cigarette smoking probably also contributed to sex differences in mortality from respiratory diseases. Cigarette smokers are more often affected than non-smokers by chronic bronchitis for example (Royal College of Physicians of London, 1962). Several studies have shown that the United Kingdom has had higher mortality and morbidity from respiratory diseases than other western countries (Chung et al., 2002; Salsiccioli et al., 2018). Salsiccioli et al. (2018) argue that between 1985 and 2015, overall respiratory disease mortality in the UK decreased for men and remained static for women. This finding fits with our result of a late twentieth century decrease in the contribution of respiratory diseases to the sex gap in mortality (Fig. 7), even though the diseases included in the respiratory group are slightly different. One potential explanation includes convergence in the rates of smoking between men and women during the observation period (Salsiccioli et al., 2018), driven by the decrease in the number of male smokers and the simultaneous increase in that of female ones.

The results point out another interesting finding: the narrowing of the gap is strongly related to the emergence of a female disadvantage from old-age diseases Fig. 6, Fig. 7. This is consistent with higher incidence rates of dementia among women than among men, which is attributed to survival to higher ages among women (Beam et al., 2018). This paper has shown that women have always survived to higher ages than men, so the question is raised about why this discrepancy in mortality from old age related causes has only recently emerged. The answer is very likely to lie in the composition of the Old age category. During the nineteenth and early twentieth centuries up to a third of deaths among people aged 55 and over were attributed to old age, and it is likely that at this time this category included deaths really due to a range of causes such as circulatory diseases and cancers, which showed a compensatory increase as mortality attributed to ‘old age’ and ‘senility’ fell (Reid et al., 2015). During this period, before the era of cigarette smoking, CVD and cancers are likely to been less differentiated by sex, and any sex-differential in senility will have been dwarfed by the presence of other causes. It is only in the last few decades that dementia has been recorded among the top causes of death, and this is possibly at least partly due to increasing longevity and better recognition and diagnosis.

It is possible to conclude that the explanations for the excess-male mortality during the first year of life at the beginning of the considered period and from neoplasms during the peak-years seem to have been found. On the contrary, the explanation for the higher male mortality from CVD is less clear, despite some contribution of cigarette smoking. Nikiforov and Mamaev (1998) after analysing several biological and social factors, conclude that: “neither the traditional single-factor hypotheses nor the multivariate approach seem to adequately explain male excess mortality from CVD” (p. 1352). However, the analysis of the secular trend of the age-cause contribution to the total sex gap in longevity represents a key tool to help identify the causes contributing to the gap.

Our study has several limitations. First, the reclassification method we used is a specific categorization designed for comparing the English and Welsh mortality experience over a long period and as such it will not be useful in a current clinical context. At the same time, a categorization used in a current clinical context would not be useful for the nineteenth century. We reclassified historic causes of death following the ICD 10 chapters, but several adjustments have been necessary. We regrouped some chapters into broader categories (e.g. “Perinatal and congenital”, “External causes”, and the residual category “Other”), and we did this because if we considered all the ICD10 chapters or groups separately our analysis would consist of too many small groups which would be difficult to analyse. Most compellingly, for much of the nineteenth and early twentieth centuries it was not possible to look at these groups separately. Moreover, as explained above, some groups of causes (specifically diarrhoea-like diseases, tuberculosis, and old age) were singled out as groups because of their burden in the nineteenth century. A second limitatation is that even after reclassifying causes of death, comparisons over time cannot be assumed always to represent real changes in mortality from particular causes. Secular trends may be affected by changes in coding practice, diagnostic fashion, and increased survival of the population (Lawlor et al., 2001; Nikiforov & Mamaev, 1998; Reid et al., 2016 b). According to Lawlor et al. (2001), changes in coding practice and increased survival should affect men and women in the same way and would therefore not explain changes in the sex differences. However, the number of deaths from a specific cause of death could be different between sexes just because it was easier to identify a specific cause for one sex rather than the other. This is the case, for example, with breast cancer. Interpretation of time trends in mortality must always consider the ways that changes and trends in diagnosis, coding and categorization may influence the results, and this is equally the case with analysis of the sex gap in mortality over time. Therefore, the results of the analysis carried out in this paper have no public health or clinical usability but they aim to provide a clearer picture of the reasons for the trend in sex-specific mortality differences over the time.

## 5 Conclusions

These findings show that the current advantage of female life expectancy in adulthood is a relatively new demographic phenomenon which has emerged since the late 19th century. This paper aimed to investigate the contribution of differences at specific age-classes and from specific causes of death to the total sex gap over this long time period.

Caution must be used when comparing causes of death over a long time-period, but results indicate that the causes of the male disadvantage have clearly shifted from the differences occurring during the first year of life to those occurring in ageing people, and from causes particularly related to biological factors to those mostly related to non-biological factors.

Further research is needed to better explain factors involved, to investigate the role of risks in labour activity for example, or factors responsible of the excess male mortality from circulatory diseases. Nevertheless, the results of the decomposition carried out in this paper provide a long-run overview of the age and cause contribution to the total sex gap in England and Wales.

## CRediT authorship contribution statement

**Valeria Maiolo:** Conceptualization, Funding acquisition, Formal analysis, Data curation, Writing - original draft, Writing - review & editing. **Alice M. Reid:** Conceptualization, Funding acquisition, Formal analysis, Data curation, Writing - original draft, Writing - review & editing.

## Declaration of competing interest

None.

## Footnotes

Supplementary data to this article can be found online at https://doi.org/10.1016/j.ssmph.2020.100584.
