# Supplementary material for: Looking for an explanation for the excessive male mortality in England and Wales since the end of the 19th century
Source: SSM Popul Health. 2020 Apr 13;11:100584. doi: 10.1016/j.ssmph.2020.100584 (PMC7178544; doi:10.1016/j.ssmph.2020.100584)
Supplement: Multimedia component 1 [file mmc1.docx]

**Appendix A**

Table A.1: Explanatory scheme of the reclassification system

Original classification system

**First step**

**Second step**

Infectious diseases

Chapter I

Certain infectious and parasitic diseases (A00-B99)

Diarrhoea**

- *A00 Cholera

- *A04 Other bacterial intestinal Infections

- *A06 Amoebiasis

- *A07 Other protozoal intestinal diseases

- *A08 Viral and other specified intestinal infections

- *A09 Other gastroenteritis and colitis of infectious and unspecified origin

- *A15-A19 Tuberculosis

Tuberculosis

Chapter II

Neoplasms (C00-D48)

Neoplasms

Other + Childbirth

Chapter III

Diseases of the blood and blood-forming organs and disorders involving the immune mechanism (D50-D89)

Other + Childbirth

Chapter IV

Endocrine, nutritional and metabolic diseases (E00-E90)

Chapter V

Mental and behavioural disorders (F00-F99)

Other + Childbirth

- *F00.1 Dementia in Alzheimer disease with late onset

- *F01 Vascular dementia

- *F02.3 Dementia in Parkinson disease

- *F03 Unspecified dementia

- *F05.1 Delirium superimposed on dementia

Old age

Chapter VI

Diseases of the nervous system (G00-G99)

Nervous system

Other + Childbirth

Chapter VII

Diseases of the eye and adnexa (H00-H59)

Other + Childbirth

Chapter VIII

Diseases of the ear and mastoid process (H60-H95)

Circulatory

Chapter IX

Diseases of the circulatory system (I00-I99)

Original classification system

Chapter X

Diseases of the respiratory system (J00-J99)

Respiratory

Digestive

Chapter XI

Diseases of the digestive system (K00-K93)

Diarrhoea***

- *K52 Other noninfective gastroenteritis and colitis

Chapter XII

Diseases of the skin and subcutaneous tissue (L00-L99)

Other + Childbirth

Chapter XIII

Diseases of the musculoskeletal system and connective tissue (M00-M99)

Other + Childbirth

Genitourinary

Chapter XIV

Diseases of the genitourinary system (N00-N99)

Other + Childbirth

Chapter XV

Pregnancy, childbirth and the puerperium (O00-O99)

Perinatal & congenital

Chapter XVI

Certain conditions originating in the perinatal period

(P00-P96)

Perinatal & congenital

Chapter XVII

Congenital malformations, deformations and chromosomal abnormalities (Q00-Q99)

Ill defined

Chapter XVIII

Symptoms, signs and abnormal clinical and laboratory findings, not elsewhere classified (R00-R99)

Old age

- *R54 Senility

External causes

Chapter XIX

Injury, poisoning and certain other consequences of external causes (S00-T98)

External causes

Chapter XX

External causes of morbidity and mortality (V01-Y98)

* Except

** From 2001 they are considered under Infectious diseases

*** From 2001 they are considered under Digestive diseases
