# Supplementary material for: Looking for an explanation for the excessive male mortality in England and Wales since the end of the 19th century
Source: SSM Popul Health. 2020 Apr 13;11:100584. doi: 10.1016/j.ssmph.2020.100584 (PMC7178544; doi:10.1016/j.ssmph.2020.100584)
Supplement: Multimedia component 3 [file mmc3.docx]

**Appendix C**

Table C.1: Age-cause-contribution in years to the total sex gap in 1881 in England and Wales

| **1881 RESULTS** | | | | | | | | | | | | | | | |
| --- | --- | --- | --- | --- | --- | --- | --- | --- | --- | --- | --- | --- | --- | --- | --- |
| Age class | Total  contribution | Genitourinary | Nervous system | Digestive | Perinatal  &  congenital | Old age | Tuberculosis | Circulatory | Neoplasms | Infectious | Diarrhoea | Respiratory | External  causes | Other + Childbirth | Ill  defined |
| <1 | **1.29669** | 0.00271 | 0.03956 | 0.04639 | 0.33079 | 0.00000 | 0.10565 | 0.00581 | 0.00041 | 0.00273 | 0.14723 | 0.31076 | 0.00288 | 0.02415 | 0.27762 |
| 1-14 | **0.21227** | 0.01127 | 0.03129 | 0.00228 | 0.00167 | 0.00000 | 0.03460 | -0.02084 | 0.00294 | -0.13377 | -0.00990 | 0.07894 | 0.16077 | 0.03059 | 0.02245 |
| 15-24 | **-0.01682** | -0.00802 | -0.00149 | -0.00267 | -0.00046 | 0.00000 | -0.07148 | -0.00627 | -0.00062 | 0.00111 | 0.00424 | 0.02033 | 0.10028 | -0.05483 | 0.00307 |
| 25-34 | **0.08037** | -0.02578 | 0.01726 | -0.00644 | -0.00035 | 0.00000 | 0.03009 | 0.00248 | -0.02115 | 0.01411 | 0.01424 | 0.05897 | 0.17893 | -0.18621 | 0.00421 |
| 35-44 | **0.33803** | -0.00637 | 0.02290 | 0.00649 | -0.00216 | 0.00000 | 0.13152 | 0.05287 | -0.09364 | 0.05810 | -0.00343 | 0.11138 | 0.17055 | -0.11945 | 0.00928 |
| 45-54 | **0.47530** | 0.01453 | 0.01817 | 0.00749 | -0.00492 | 0.00022 | 0.14528 | 0.01563 | -0.11264 | 0.03540 | 0.00333 | 0.17864 | 0.13623 | 0.02784 | 0.01009 |
| 55-64 | **0.36337** | 0.02714 | 0.03489 | 0.01198 | -0.00349 | -0.00307 | 0.08581 | 0.01061 | -0.06981 | 0.02073 | 0.00218 | 0.12804 | 0.08946 | 0.02261 | 0.00629 |
| 65-74 | **0.23109** | 0.05265 | 0.03137 | 0.00868 | 0.00070 | -0.00075 | 0.02019 | 0.02716 | -0.02499 | 0.01244 | 0.00041 | 0.05165 | 0.03311 | 0.01579 | 0.00268 |
| 75+ | **0.12971** | 0.03907 | 0.01395 | 0.00067 | 0.00106 | 0.01502 | 0.00241 | 0.02982 | -0.00550 | 0.00450 | 0.00446 | 0.01764 | 0.00070 | 0.00642 | -0.00051 |
| Total | **3.11001** | **0.10721** | **0.20790** | **0.07487** | **0.32283** | **0.01143** | **0.48407** | **0.11727** | **-0.32501** | **0.01534** | **0.16278** | **0.95634** | **0.87291** | **-0.23311** | **0.33517** |

Source: Author computations using data from the Human Mortality Database and from Davenport, R. J. (2007), Annual deaths by cause, age and sex in England and Wales, 1848-1900. *Economic and Social Data Services* (SN5705). http://doi.org/10.5255/UKDA-SN-5705-1.

Note: Negative numbers mean that mortality is lower in that age group for males than females, and thus acts to offset the female advantage at other age groups.

Table C.2: Age-cause-contribution in years to the total sex gap in 1891 in England and Wales

| **1891 RESULTS** | | | | | | | | | | | | | | | |
| --- | --- | --- | --- | --- | --- | --- | --- | --- | --- | --- | --- | --- | --- | --- | --- |
| Age class | Total contribution | Genitourinary | Nervous system | Digestive | Perinatal  &  congenital | Old age | Tuberculosis | Circulatory | Neoplasms | Infectious | Diarrhoea | Respiratory | External  causes | Other + Childbirth | Ill  defined |
| <1 | **1.55745** | 0.00401 | 0.04847 | 0.04731 | 0.41531 | 0.00000 | 0.09656 | 0.00241 | -0.00064 | -0.00690 | 0.15373 | 0.45426 | -0.00178 | 0.03889 | 0.30582 |
| 1-14 | **0.15346** | 0.00711 | 0.04419 | 0.01029 | -0.00268 | 0.00000 | 0.00673 | -0.02002 | 0.00260 | -0.13527 | -0.00894 | 0.08372 | 0.13192 | 0.01624 | 0.01758 |
| 15-24 | **0.05560** | -0.01120 | 0.00691 | -0.02188 | -0.00031 | 0.00000 | -0.04200 | -0.02050 | 0.00359 | -0.00447 | 0.03087 | 0.06911 | 0.16044 | -0.11733 | 0.00236 |
| 25-34 | **0.17071** | -0.01641 | 0.00317 | -0.00630 | -0.00003 | 0.00000 | 0.09896 | 0.00110 | -0.01635 | 0.00966 | 0.01599 | 0.12792 | 0.15289 | -0.20372 | 0.00385 |
| 35-44 | **0.36515** | -0.01175 | 0.00622 | -0.00114 | -0.00020 | 0.00000 | 0.16806 | -0.01045 | -0.08062 | 0.03488 | 0.00506 | 0.21388 | 0.14363 | -0.10923 | 0.00681 |
| 45-54 | **0.51210** | 0.01417 | 0.01389 | 0.00837 | -0.00113 | -0.00007 | 0.16212 | 0.00125 | -0.11641 | 0.02386 | 0.00496 | 0.25876 | 0.11260 | 0.01873 | 0.01101 |
| 55-64 | **0.38989** | 0.04099 | 0.01879 | 0.01405 | 0.00004 | -0.00284 | 0.07107 | 0.01643 | -0.06080 | 0.01454 | 0.00036 | 0.18710 | 0.06700 | 0.01634 | 0.00683 |
| 65-74 | **0.20259** | 0.04532 | 0.01967 | 0.00364 | -0.00167 | -0.00933 | 0.01834 | 0.02828 | -0.01725 | 0.00607 | -0.00209 | 0.06524 | 0.02840 | 0.01411 | 0.00388 |
| 75+ | **0.08307** | 0.03006 | 0.00914 | 0.00172 | 0.00006 | -0.00013 | 0.00005 | 0.02282 | -0.00359 | 0.00156 | 0.00211 | 0.01075 | 0.00236 | 0.00371 | 0.00247 |
| Total | **3.49002** | **0.10228** | **0.17044** | **0.05604** | **0.40939** | **-0.01236** | **0.57988** | **0.02134** | **-0.28946** | **-0.05608** | **0.20205** | **1.47073** | **0.79744** | **-0.32227** | **0.36060** |

Source: Author computations using data from the Human Mortality Database and from Davenport, R. J. (2007), Annual deaths by cause, age and sex in England and Wales, 1848-1900. *Economic and Social Data Services* (SN5705). http://doi.org/10.5255/UKDA-SN-5705-1.

Note: Negative numbers mean that mortality is lower in that age group for males than females, and thus acts to offset the female advantage at other age groups.

Table C.3: Age-cause-contribution in years to the total sex gap in 1901 in England and Wales

| **1901 RESULTS** | | | | | | | | | | | | | | | |
| --- | --- | --- | --- | --- | --- | --- | --- | --- | --- | --- | --- | --- | --- | --- | --- |
| Age class | Total contribution | Genitourinary | Nervous system | Digestive | Perinatal  &  congenital | Old age | Tuberculosis | Circulatory | Neoplasms | Infectious | Diarrhoea | Respiratory | External causes | Other + Childbirth | Ill  defined |
| <1 | **1.57456** | 0.00968 | 0.03806 | 0.04928 | 0.53849 | 0.00000 | 0.07517 | 0.00681 | 0.00008 | 0.02261 | 0.29017 | 0.30411 | 0.00531 | 0.02972 | 0.20507 |
| 1-14 | **0.11626** | 0.01173 | 0.01985 | 0.00769 | 0.00861 | 0.00000 | -0.01418 | -0.03012 | 0.00580 | -0.08056 | 0.01951 | 0.04581 | 0.09852 | 0.02116 | 0.00245 |
| 15-24 | **0.16792** | -0.00333 | 0.00484 | -0.01940 | 0.00094 | 0.00000 | 0.02236 | -0.00363 | 0.00049 | 0.01155 | 0.03439 | 0.05462 | 0.14730 | -0.08234 | 0.00012 |
| 25-34 | **0.22233** | -0.01763 | 0.00568 | -0.01177 | 0.00036 | 0.00000 | 0.13187 | 0.01410 | -0.02175 | 0.02411 | 0.03010 | 0.08335 | 0.15483 | -0.17270 | 0.00177 |
| 35-44 | **0.33856** | -0.01528 | 0.00705 | -0.00588 | -0.00049 | 0.00000 | 0.19189 | 0.00510 | -0.08341 | 0.06093 | 0.01509 | 0.12267 | 0.14784 | -0.11034 | 0.00339 |
| 45-54 | **0.49918** | 0.01965 | 0.00663 | 0.00224 | -0.00120 | 0.00000 | 0.20383 | 0.01065 | -0.11936 | 0.04797 | 0.00747 | 0.17048 | 0.12968 | 0.01639 | 0.00476 |
| 55-64 | **0.46875** | 0.05825 | 0.01604 | 0.01926 | 0.00073 | -0.00451 | 0.09647 | 0.03175 | -0.04655 | 0.03037 | 0.00241 | 0.14511 | 0.08897 | 0.02339 | 0.00705 |
| 65-74 | **0.31168** | 0.07607 | 0.02304 | 0.00870 | -0.00018 | 0.01574 | 0.02134 | 0.05221 | -0.00943 | 0.01355 | 0.00152 | 0.05098 | 0.03439 | 0.01977 | 0.00400 |
| 75+ | **0.14075** | 0.04807 | 0.01213 | 0.00282 | 0.00005 | 0.01931 | 0.00223 | 0.02914 | -0.00532 | 0.00432 | 0.00110 | 0.00683 | 0.00405 | 0.01528 | 0.00074 |
| Total | **3.83999** | **0.18722** | **0.13332** | **0.05293** | **0.54730** | **0.03054** | **0.73099** | **0.11601** | **-0.27945** | **0.13485** | **0.40176** | **0.98396** | **0.81090** | **-0.23967** | **0.22936** |

Source: Author computations using data from the Human Mortality Database and from the Office for National Statistics (nd b), The 20th Century Mortality Files, 1901-2000 release. This is available at: [https://webarchive.nationalarchives.gov.uk/20150908090558/http://www.ons.gov.uk/ons/publications/re-reference-tables.html?edition=tcm%3A77-215593](https://webarchive.nationalarchives.gov.uk/20150908090558/http:/www.ons.gov.uk/ons/publications/re-reference-tables.html?edition=tcm%3A77-215593)

Note: Negative numbers mean that mortality is lower in that age group for males than females, and thus acts to offset the female advantage at other age groups.

Table C.4: Age-cause-contribution in years to the total sex gap in 1911 in England and Wales

| **1911 RESULTS** | | | | | | | | | | | | | | | |
| --- | --- | --- | --- | --- | --- | --- | --- | --- | --- | --- | --- | --- | --- | --- | --- |
| Age class | Total contribution | Genitourinary | Nervous system | Digestive | Perinatal &  congenital | Old age | Tuberculosis | Circulatory | Neoplasms | Infectious | Diarrhoea | Respiratory | External  causes | Other + Childbirth | Ill  defined |
| <1 | **1.41158** | 0.00056 | 0.02430 | 0.04788 | 0.52080 | 0.00000 | 0.03989 | 0.00171 | 0.00006 | 0.01036 | 0.33567 | 0.24797 | 0.00515 | 0.02144 | 0.15578 |
| 1-14 | **0.18081** | -0.00082 | 0.02678 | 0.01980 | 0.00229 | 0.00000 | -0.02632 | -0.02653 | 0.01272 | -0.04658 | 0.00202 | 0.09341 | 0.10990 | 0.01199 | 0.00215 |
| 15-24 | **0.15775** | -0.00290 | 0.01214 | 0.01328 | 0.00057 | 0.00000 | -0.01259 | -0.02535 | 0.00540 | 0.00975 | 0.01337 | 0.05086 | 0.14879 | -0.05587 | 0.00031 |
| 25-34 | **0.23600** | -0.00053 | 0.00837 | 0.00779 | -0.00035 | 0.00000 | 0.10678 | -0.00831 | -0.01611 | 0.02672 | 0.02003 | 0.08804 | 0.15861 | -0.15435 | -0.00068 |
| 35-44 | **0.30083** | -0.00079 | 0.00116 | 0.00354 | 0.00003 | 0.00000 | 0.15516 | 0.01304 | -0.09088 | 0.06181 | 0.00873 | 0.10430 | 0.14528 | -0.10083 | 0.00029 |
| 45-54 | **0.45841** | 0.03206 | 0.00159 | 0.01215 | -0.00029 | -0.00025 | 0.16758 | 0.00740 | -0.08284 | 0.05921 | 0.00611 | 0.13960 | 0.12384 | -0.01185 | 0.00411 |
| 55-64 | **0.52106** | 0.07136 | 0.01255 | 0.01636 | -0.00006 | -0.00080 | 0.09500 | 0.09237 | -0.01857 | 0.03511 | 0.00470 | 0.12795 | 0.08632 | -0.00809 | 0.00685 |
| 65-74 | **0.43624** | 0.09632 | 0.01284 | 0.01008 | 0.00000 | 0.02230 | 0.02516 | 0.11063 | 0.01806 | 0.01560 | 0.00427 | 0.07726 | 0.03250 | 0.00699 | 0.00423 |
| 75+ | **0.21731** | 0.07000 | 0.00573 | 0.00036 | 0.00000 | 0.04078 | 0.00213 | 0.05124 | 0.00155 | 0.00476 | 0.00464 | 0.02625 | 0.00671 | 0.00268 | 0.00048 |
| Total | **3.91999** | **0.26526** | **0.10545** | **0.13124** | **0.52298** | **0.06203** | **0.55279** | **0.21619** | **-0.17060** | **0.17672** | **0.39953** | **0.95565** | **0.81710** | **-0.28788** | **0.17352** |

Source: Author computations using data from the Human Mortality Database and from the Office for National Statistics (nd b), The 20th Century Mortality Files, 1901-2000 release. This is available at: [https://webarchive.nationalarchives.gov.uk/20150908090558/http://www.ons.gov.uk/ons/publications/re-reference-tables.html?edition=tcm%3A77-215593](https://webarchive.nationalarchives.gov.uk/20150908090558/http:/www.ons.gov.uk/ons/publications/re-reference-tables.html?edition=tcm%3A77-215593)

Note: Negative numbers mean that mortality is lower in that age group for males than females, and thus acts to offset the female advantage at other age groups.

Table C.5: Age-cause-contribution in years to the total sex gap in 1921 in England and Wales

| **1921 RESULTS** | | | | | | | | | | | | | | | |
| --- | --- | --- | --- | --- | --- | --- | --- | --- | --- | --- | --- | --- | --- | --- | --- |
| Age class | Total contribution | Genitourinary | Nervous system | Digestive | Perinatal &  congenital | Old age | Tuberculosis | Circulatory | Neoplasms | Infectious | Diarrhoea | Respiratory | External  causes | Other + Childbirth | Ill  defined |
| <1 | **1.24461** | 0.00675 | 0.00826 | 0.04966 | 0.50518 | 0.00000 | 0.01982 | 0.00329 | 0.00163 | 0.01374 | 0.25252 | 0.24195 | 0.00906 | 0.02428 | 0.10848 |
| 1-14 | **0.18849** | 0.00478 | 0.01399 | 0.01671 | 0.00268 | 0.00000 | -0.02301 | -0.02105 | 0.01166 | -0.06202 | 0.02325 | 0.09356 | 0.11298 | 0.00989 | 0.00507 |
| 15-24 | **0.10150** | -0.00635 | 0.01059 | 0.02973 | 0.00020 | 0.00000 | -0.11261 | 0.00283 | 0.01222 | 0.00502 | 0.00340 | 0.06784 | 0.15304 | -0.06519 | 0.00078 |
| 25-34 | **0.16646** | -0.00114 | 0.00818 | 0.00987 | 0.00051 | 0.00000 | 0.06070 | 0.05644 | -0.01463 | 0.01721 | -0.00009 | 0.07488 | 0.10274 | -0.14936 | 0.00115 |
| 35-44 | **0.31064** | -0.00545 | -0.00035 | 0.01690 | -0.00025 | 0.00000 | 0.16303 | 0.02868 | -0.08881 | 0.06071 | 0.00189 | 0.14060 | 0.10732 | -0.11576 | 0.00212 |
| 45-54 | **0.45015** | 0.02380 | -0.00032 | 0.01529 | 0.00011 | 0.00010 | 0.15845 | 0.03224 | -0.09743 | 0.05503 | 0.00131 | 0.17068 | 0.10981 | -0.02193 | 0.00302 |
| 55-64 | **0.62197** | 0.06498 | 0.01392 | 0.01683 | 0.00003 | 0.00079 | 0.08688 | 0.11235 | 0.03116 | 0.05375 | 0.00037 | 0.16552 | 0.09133 | -0.01943 | 0.00349 |
| 65-74 | **0.55823** | 0.10181 | 0.01624 | 0.00169 | 0.00006 | 0.02426 | 0.02067 | 0.15635 | 0.06790 | 0.02095 | -0.00051 | 0.11518 | 0.04307 | -0.01209 | 0.00265 |
| 75+ | **0.30795** | 0.10725 | 0.00658 | 0.00145 | 0.00000 | 0.02191 | 0.00030 | 0.11197 | 0.01559 | 0.00444 | 0.00108 | 0.03141 | 0.00783 | -0.00266 | 0.00081 |
| Total | **3.95000** | **0.29643** | **0.07708** | **0.15813** | **0.50853** | **0.04706** | **0.37422** | **0.48310** | **-0.06072** | **0.16882** | **0.28322** | **1.10163** | **0.73718** | **-0.35226** | **0.12759** |

Source: Author computations using data from the Human Mortality Database and from the Office for National Statistics (nd b), The 20th Century Mortality Files, 1901-2000 release. This is available at: [https://webarchive.nationalarchives.gov.uk/20150908090558/http://www.ons.gov.uk/ons/publications/re-reference-tables.html?edition=tcm%3A77-215593](https://webarchive.nationalarchives.gov.uk/20150908090558/http:/www.ons.gov.uk/ons/publications/re-reference-tables.html?edition=tcm%3A77-215593)

Note: Negative numbers mean that mortality is lower in that age group for males than females, and thus acts to offset the female advantage at other age groups.

Table C.6: Age-cause-contribution in years to the total sex gap in 1931 in England and Wales

| **1931 RESULTS** | | | | | | | | | | | | | | | |
| --- | --- | --- | --- | --- | --- | --- | --- | --- | --- | --- | --- | --- | --- | --- | --- |
| Age class | Total contribution | Genitourinary | Nervous system | Digestive | Perinatal &  congenital | Old age | Tuberculosis | Circulatory | Neoplasms | Infectious | Diarrhoea | Respiratory | External  causes | Other + Childbirth | Ill  defined |
| <1 | **1.15993** | 0.00461 | 0.00989 | 0.03570 | 0.55581 | 0.00000 | 0.01581 | 0.00082 | 0.00190 | 0.01080 | 0.12050 | 0.29509 | 0.00695 | 0.04041 | 0.06166 |
| 1-14 | **0.31991** | -0.00153 | 0.02834 | 0.02334 | 0.00555 | -0.00019 | 0.00014 | -0.01980 | 0.01656 | -0.01399 | 0.00140 | 0.10301 | 0.13598 | 0.03703 | 0.00408 |
| 15-24 | **0.12582** | -0.00245 | 0.02800 | 0.01987 | 0.00125 | -0.00009 | -0.09196 | -0.02431 | 0.00929 | 0.00787 | 0.00175 | 0.03955 | 0.18503 | -0.04835 | 0.00037 |
| 25-34 | **0.06801** | -0.00883 | 0.00509 | 0.02317 | 0.00039 | -0.00013 | 0.02682 | -0.02651 | -0.00751 | 0.00809 | 0.00055 | 0.04281 | 0.12969 | -0.12556 | -0.00006 |
| 35-44 | **0.30750** | -0.00770 | 0.00918 | 0.04481 | 0.00028 | -0.00005 | 0.15136 | 0.00816 | -0.08398 | 0.03623 | -0.00010 | 0.11992 | 0.11193 | -0.08350 | 0.00095 |
| 45-54 | **0.54406** | 0.02472 | 0.00322 | 0.05708 | 0.00005 | -0.00051 | 0.17007 | 0.07294 | -0.09342 | 0.04532 | 0.00058 | 0.18797 | 0.11047 | -0.03661 | 0.00219 |
| 55-64 | **0.63309** | 0.06622 | 0.00692 | 0.03582 | 0.00002 | -0.00109 | 0.08537 | 0.17981 | 0.05229 | 0.03588 | 0.00058 | 0.12883 | 0.08493 | -0.04407 | 0.00158 |
| 65-74 | **0.65502** | 0.12395 | 0.01241 | 0.02882 | -0.00002 | 0.00483 | 0.01905 | 0.25547 | 0.12125 | 0.01417 | 0.00005 | 0.06522 | 0.03439 | -0.02618 | 0.00161 |
| 75+ | **0.34665** | 0.12016 | 0.00746 | 0.00732 | 0.00005 | -0.00249 | 0.00118 | 0.16197 | 0.02980 | 0.00342 | 0.00002 | 0.02092 | 0.00514 | -0.00908 | 0.00078 |
| Total | **4.15999** | **0.31914** | **0.11051** | **0.27594** | **0.56338** | **0.00028** | **0.37783** | **0.60855** | **0.04618** | **0.14778** | **0.12534** | **1.00332** | **0.80450** | **-0.29590** | **0.07314** |

Source: Author computations using data from the Human Mortality Database and from the Office for National Statistics (nd b), The 20th Century Mortality Files, 1901-2000 release. This is available at: [https://webarchive.nationalarchives.gov.uk/20150908090558/http://www.ons.gov.uk/ons/publications/re-reference-tables.html?edition=tcm%3A77-215593](https://webarchive.nationalarchives.gov.uk/20150908090558/http:/www.ons.gov.uk/ons/publications/re-reference-tables.html?edition=tcm%3A77-215593)

Note: Negative numbers mean that mortality is lower in that age group for males than females, and thus acts to offset the female advantage at other age groups.

Table C.7: Age-cause-contribution in years to the total sex gap in 1939 in England and Wales

| **1939 RESULTS** | | | | | | | | | | | | | | | |
| --- | --- | --- | --- | --- | --- | --- | --- | --- | --- | --- | --- | --- | --- | --- | --- |
| Age class | Total contribution | Genitourinary | Nervous system | Digestive | Perinatal &  congenital | Old age | Tuberculosis | Circulatory | Neoplasms | Infectious | Diarrhoea | Respiratory | External  causes | Other + Childbirth | Ill  defined |
| <1 | **0.80929** | 0.00727 | 0.00871 | 0.03004 | 0.44770 | 0.00000 | 0.00712 | 0.00052 | -0.00176 | -0.00204 | 0.08245 | 0.14830 | 0.01675 | 0.03518 | 0.02905 |
| 1-14 | **0.23242** | 0.00116 | 0.01510 | 0.02014 | -0.00564 | 0.00000 | 0.00138 | -0.00678 | 0.01379 | -0.00051 | 0.00415 | 0.02281 | 0.14892 | 0.01661 | 0.00130 |
| 15-24 | **0.17059** | 0.00596 | 0.02880 | 0.03126 | 0.00190 | 0.00047 | -0.16207 | -0.03080 | 0.01226 | 0.00240 | -0.00011 | 0.02598 | 0.30820 | -0.05368 | 0.00002 |
| 25-34 | **0.10946** | -0.00144 | 0.00312 | 0.02294 | 0.00043 | 0.00061 | 0.01831 | -0.02380 | -0.00311 | 0.00671 | 0.00156 | 0.01332 | 0.21242 | -0.14190 | 0.00031 |
| 35-44 | **0.27440** | -0.00914 | 0.01076 | 0.05253 | 0.00079 | 0.00174 | 0.12960 | 0.00772 | -0.07904 | 0.01330 | 0.00125 | 0.06954 | 0.13327 | -0.05903 | 0.00111 |
| 45-54 | **0.61660** | 0.01184 | 0.00055 | 0.06821 | -0.00045 | 0.00007 | 0.15993 | 0.18415 | -0.07555 | 0.03099 | 0.00089 | 0.16252 | 0.11118 | -0.03842 | 0.00070 |
| 55-64 | **0.92870** | 0.05963 | 0.00854 | 0.06439 | 0.00014 | 0.00017 | 0.10263 | 0.36621 | 0.07600 | 0.03270 | 0.00090 | 0.16204 | 0.09994 | -0.04527 | 0.00069 |
| 65-74 | **0.77330** | 0.11685 | 0.01095 | 0.02563 | -0.00004 | 0.00124 | 0.02498 | 0.36566 | 0.13195 | 0.01522 | 0.00028 | 0.08140 | 0.04147 | -0.04349 | 0.00120 |
| 75+ | **0.49526** | 0.13011 | 0.00656 | 0.01267 | 0.00000 | 0.00739 | 0.00194 | 0.24351 | 0.05940 | 0.00357 | 0.00024 | 0.03194 | 0.00708 | -0.00948 | 0.00034 |
| Total | **4.41002** | **0.32224** | **0.09308** | **0.32781** | **0.44482** | **0.01167** | **0.28382** | **1.10640** | **0.13393** | **0.10233** | **0.09160** | **0.71785** | **1.07923** | **-0.33948** | **0.03472** |

Source: Author computations using data from the Human Mortality Database and from the Office for National Statistics (nd b), The 20th Century Mortality Files, 1901-2000 release. This is available at: [https://webarchive.nationalarchives.gov.uk/20150908090558/http://www.ons.gov.uk/ons/publications/re-reference-tables.html?edition=tcm%3A77-215593](https://webarchive.nationalarchives.gov.uk/20150908090558/http:/www.ons.gov.uk/ons/publications/re-reference-tables.html?edition=tcm%3A77-215593)

Note: Negative numbers mean that mortality is lower in that age group for males than females, and thus acts to offset the female advantage at other age groups.

Table C.8: Age-cause-contribution in years to the total sex gap in 1951 in England and Wales

| **1951 RESULTS** | | | | | | | | | | | | | | | |
| --- | --- | --- | --- | --- | --- | --- | --- | --- | --- | --- | --- | --- | --- | --- | --- |
| Age class | Total contribution | Genitourinary | Nervous system | Digestive | Perinatal &  congenital | Old age | Tuberculosis | Circulatory | Neoplasms | Infectious | Diarrhoea | Respiratory | External  causes | Other + Childbirth | Ill  defined |
| <1 | **0.55802** | 0.00162 | 0.00815 | 0.01016 | 0.40299 | 0.00000 | 0.00248 | 0.00179 | 0.00137 | -0.00065 | 0.02543 | 0.06849 | 0.02354 | 0.00959 | 0.00306 |
| 1-14 | **0.16377** | -0.00045 | 0.00435 | 0.01306 | 0.01161 | 0.00000 | -0.00138 | -0.00316 | 0.01678 | 0.00255 | 0.00369 | -0.00064 | 0.11031 | 0.00674 | 0.00032 |
| 15-24 | **0.18403** | 0.00325 | 0.00402 | 0.00573 | 0.00632 | 0.00000 | -0.04644 | -0.00128 | 0.01215 | 0.00432 | 0.00020 | 0.00582 | 0.20454 | -0.01536 | 0.00076 |
| 25-34 | **0.11749** | 0.00538 | 0.00480 | 0.00655 | 0.00063 | 0.00000 | -0.01663 | 0.00400 | -0.00374 | 0.00072 | 0.00063 | -0.00231 | 0.14932 | -0.03212 | 0.00026 |
| 35-44 | **0.18836** | 0.00024 | -0.00075 | 0.02590 | 0.00039 | -0.00009 | 0.04125 | 0.05913 | -0.03808 | 0.00403 | 0.00020 | 0.02877 | 0.08937 | -0.02211 | 0.00011 |
| 45-54 | **0.69291** | 0.00523 | -0.00204 | 0.05305 | 0.00003 | -0.00007 | 0.09371 | 0.24363 | 0.05181 | 0.00416 | 0.00063 | 0.17898 | 0.07614 | -0.01387 | 0.00152 |
| 55-64 | **1.38131** | 0.02679 | 0.00301 | 0.06174 | 0.00004 | -0.00033 | 0.10068 | 0.51121 | 0.22741 | 0.01408 | 0.00068 | 0.39427 | 0.06018 | -0.01949 | 0.00106 |
| 65-74 | **1.23812** | 0.07476 | 0.00406 | 0.04978 | -0.00024 | 0.00117 | 0.04379 | 0.51227 | 0.23446 | 0.00878 | -0.00039 | 0.30805 | 0.02779 | -0.02675 | 0.00066 |
| 75+ | **0.59601** | 0.11627 | 0.00158 | 0.01559 | 0.00032 | -0.00625 | 0.00684 | 0.23983 | 0.11117 | 0.00346 | -0.00037 | 0.12164 | -0.00151 | -0.01342 | 0.00088 |
| Total | **5.12002** | **0.23300** | **0.02717** | **0.24154** | **0.42209** | **-0.00557** | **0.22429** | **1.56741** | **0.61333** | **0.04146** | **0.03069** | **1.10307** | **0.73969** | **-0.12678** | **0.00863** |

Source: Author computations using data from the Human Mortality Database and from the Office for National Statistics (nd b), The 20th Century Mortality Files, 1901-2000 release. This is available at: [https://webarchive.nationalarchives.gov.uk/20150908090558/http://www.ons.gov.uk/ons/publications/re-reference-tables.html?edition=tcm%3A77-215593](https://webarchive.nationalarchives.gov.uk/20150908090558/http:/www.ons.gov.uk/ons/publications/re-reference-tables.html?edition=tcm%3A77-215593)

Note: Negative numbers mean that mortality is lower in that age group for males than females, and thus acts to offset the female advantage at other age groups.

Table C.9: Age-cause-contribution in years to the total sex gap in 1961 in England and Wales

| **1961 RESULTS** | | | | | | | | | | | | | | | |
| --- | --- | --- | --- | --- | --- | --- | --- | --- | --- | --- | --- | --- | --- | --- | --- |
| Age class | Total contribution | Genitourinary | Nervous system | Digestive | Perinatal &  congenital | Old age | Tuberculosis | Circulatory | Neoplasms | Infectious | Diarrhoea | Respiratory | External  causes | Other + Childbirth | Ill  defined |
| <1 | **0.37146** | 0.00363 | 0.00300 | 0.01207 | 0.28642 | 0.00000 | -0.00042 | 0.00380 | 0.00299 | 0.00146 | 0.00121 | 0.03864 | 0.01458 | 0.00268 | 0.00139 |
| 1-14 | **0.15611** | -0.00151 | 0.00398 | 0.00033 | 0.00632 | 0.00000 | -0.00082 | 0.00488 | 0.01130 | 0.00889 | 0.00723 | 0.01628 | 0.09775 | 0.00152 | -0.00005 |
| 15-24 | **0.28946** | 0.00020 | 0.00629 | 0.00243 | 0.00190 | 0.00000 | -0.00071 | 0.00615 | 0.01759 | 0.00096 | 0.00050 | 0.00361 | 0.25698 | -0.00626 | -0.00017 |
| 25-34 | **0.17110** | 0.00519 | 0.00061 | 0.00622 | 0.00069 | 0.00000 | -0.00254 | 0.02824 | 0.00651 | 0.00074 | 0.00041 | -0.00242 | 0.14001 | -0.01337 | 0.00082 |
| 35-44 | **0.22296** | 0.00559 | -0.00394 | 0.01136 | 0.00001 | 0.00000 | 0.00718 | 0.13965 | -0.05189 | 0.00158 | -0.00010 | 0.01522 | 0.10434 | -0.00671 | 0.00066 |
| 45-54 | **0.62681** | 0.00635 | -0.00047 | 0.02409 | 0.00278 | 0.00000 | 0.01685 | 0.38957 | 0.02504 | -0.00041 | -0.00017 | 0.09918 | 0.06429 | -0.00054 | 0.00023 |
| 55-64 | **1.57055** | 0.01334 | 0.00432 | 0.04181 | -0.00029 | -0.00011 | 0.03116 | 0.74700 | 0.34282 | 0.00210 | 0.00027 | 0.34541 | 0.05335 | -0.01137 | 0.00074 |
| 65-74 | **1.59354** | 0.04273 | 0.00487 | 0.03808 | 0.00064 | 0.00035 | 0.02757 | 0.67515 | 0.37837 | 0.00212 | -0.00046 | 0.42646 | 0.02134 | -0.02443 | 0.00074 |
| 75+ | **0.85802** | 0.09452 | 0.00130 | 0.02470 | 0.00047 | -0.01148 | 0.00827 | 0.29009 | 0.19019 | 0.00039 | -0.00082 | 0.27363 | 0.00207 | -0.01647 | 0.00117 |
| Total | **5.86001** | **0.17004** | **0.01995** | **0.16109** | **0.29895** | **-0.01124** | **0.08655** | **2.28453** | **0.92291** | **0.01784** | **0.00807** | **1.21601** | **0.75472** | **-0.07494** | **0.00552** |

Source: Author computations using data from the Human Mortality Database and from the Office for National Statistics (nd b), The 20th Century Mortality Files, 1901-2000 release. This is available at: [https://webarchive.nationalarchives.gov.uk/20150908090558/http://www.ons.gov.uk/ons/publications/re-reference-tables.html?edition=tcm%3A77-215593](https://webarchive.nationalarchives.gov.uk/20150908090558/http:/www.ons.gov.uk/ons/publications/re-reference-tables.html?edition=tcm%3A77-215593)

Note: Negative numbers mean that mortality is lower in that age group for males than females, and thus acts to offset the female advantage at other age groups.

Table C.10: Age-cause-contribution in years to the total sex gap in 1971 in England and Wales

| **1971 RESULTS** | | | | | | | | | | | | | | | |
| --- | --- | --- | --- | --- | --- | --- | --- | --- | --- | --- | --- | --- | --- | --- | --- |
| Age class | Total contribution | Genitourinary | Nervous system | Digestive | Perinatal &  congenital | Old age | Tuberculosis | Circulatory | Neoplasms | Infectious | Diarrhoea | Respiratory | External  causes | Other + Childbirth | Ill  defined |
| <1 | **0.34476** | 0.00328 | 0.00315 | 0.00473 | 0.24560 | 0.00000 | 0.00000 | 0.00150 | -0.00070 | 0.00307 | 0.01026 | 0.05688 | 0.00610 | 0.00048 | 0.01042 |
| 1-14 | **0.13396** | 0.00038 | 0.00791 | 0.00371 | 0.00635 | 0.00000 | -0.00059 | 0.00226 | 0.01350 | -0.00331 | -0.00362 | 0.00253 | 0.09977 | 0.00424 | 0.00083 |
| 15-24 | **0.27110** | 0.00087 | 0.01311 | 0.00289 | 0.00449 | 0.00000 | -0.00049 | 0.00654 | 0.01696 | 0.00120 | -0.00001 | 0.01024 | 0.21900 | -0.00472 | 0.00102 |
| 25-34 | **0.14954** | 0.00045 | 0.00425 | 0.00224 | 0.00241 | 0.00000 | 0.00112 | 0.03128 | -0.00037 | 0.00110 | 0.00012 | 0.00269 | 0.11166 | -0.00689 | -0.00042 |
| 35-44 | **0.23525** | -0.00098 | 0.00247 | 0.00855 | 0.00240 | 0.00011 | 0.00198 | 0.19112 | -0.05717 | 0.00245 | -0.00012 | 0.00843 | 0.08460 | -0.00845 | -0.00013 |
| 45-54 | **0.66214** | 0.00134 | -0.00150 | 0.01261 | 0.00117 | -0.00038 | 0.00445 | 0.57461 | -0.02967 | 0.00055 | 0.00026 | 0.05215 | 0.05115 | -0.00457 | -0.00004 |
| 55-64 | **1.45970** | 0.00739 | 0.00309 | 0.02219 | 0.00052 | -0.00055 | 0.01023 | 0.87871 | 0.30201 | 0.00212 | -0.00049 | 0.20268 | 0.03776 | -0.00638 | 0.00043 |
| 65-74 | **1.86588** | 0.01712 | 0.00950 | 0.02760 | 0.00113 | 0.00048 | 0.01120 | 0.91405 | 0.50633 | 0.00144 | -0.00013 | 0.37782 | 0.01529 | -0.01624 | 0.00031 |
| 75+ | **1.15765** | 0.05174 | 0.00499 | 0.02322 | 0.00027 | -0.01240 | 0.00694 | 0.38661 | 0.32370 | 0.00013 | -0.00007 | 0.40680 | -0.01299 | -0.02180 | 0.00052 |
| Total | **6.27998** | **0.08158** | **0.04698** | **0.10774** | **0.26434** | **-0.01275** | **0.03485** | **2.98668** | **1.07459** | **0.00874** | **0.00621** | **1.12022** | **0.61233** | **-0.06435** | **0.01293** |

Source: Author computations using data from the Human Mortality Database and from the Office for National Statistics (nd b), The 20th Century Mortality Files, 1901-2000 release. This is available at: [https://webarchive.nationalarchives.gov.uk/20150908090558/http://www.ons.gov.uk/ons/publications/re-reference-tables.html?edition=tcm%3A77-215593](https://webarchive.nationalarchives.gov.uk/20150908090558/http:/www.ons.gov.uk/ons/publications/re-reference-tables.html?edition=tcm%3A77-215593)

Note: Negative numbers mean that mortality is lower in that age group for males than females, and thus acts to offset the female advantage at other age groups.

Table C.11: Age-cause-contribution in years to the total sex gap in 1981 in England and Wales

| **1981 RESULTS** | | | | | | | | | | | | | | | |
| --- | --- | --- | --- | --- | --- | --- | --- | --- | --- | --- | --- | --- | --- | --- | --- |
| Age class | Total contribution | Genitourinary | Nervous system | Digestive | Perinatal &  congenital | Old age | Tuberculosis | Circulatory | Neoplasms | Infectious | Diarrhoea | Respiratory | External  causes | Other + Childbirth | Ill  defined |
| <1 | **0.24368** | 0.00255 | 0.00094 | 0.00284 | 0.15793 | 0.00000 | 0.00000 | 0.00245 | -0.00002 | 0.00629 | 0.00136 | 0.03119 | 0.00250 | -0.00324 | 0.03889 |
| 1-14 | **0.07405** | -0.00071 | 0.00271 | 0.00113 | -0.00138 | 0.00000 | 0.00016 | 0.00316 | 0.00718 | 0.00314 | 0.00014 | 0.00482 | 0.05314 | -0.00074 | 0.00131 |
| 15-24 | **0.26784** | -0.00050 | 0.00826 | -0.00060 | -0.00089 | 0.00000 | 0.00026 | 0.00615 | 0.01358 | 0.00097 | -0.00028 | 0.00690 | 0.23370 | 0.00034 | -0.00005 |
| 25-34 | **0.16417** | -0.00080 | 0.00413 | 0.00221 | 0.00328 | 0.00000 | 0.00023 | 0.02743 | -0.01613 | 0.00070 | 0.00012 | 0.00648 | 0.13676 | 0.00004 | -0.00026 |
| 35-44 | **0.20581** | -0.00057 | 0.00080 | 0.00903 | 0.00076 | -0.00012 | 0.00226 | 0.17090 | -0.07420 | 0.00139 | 0.00000 | 0.00192 | 0.09014 | 0.00379 | -0.00027 |
| 45-54 | **0.55138** | -0.00213 | 0.00004 | 0.01309 | 0.00087 | -0.00009 | 0.00224 | 0.52109 | -0.06029 | 0.00234 | -0.00026 | 0.01752 | 0.05227 | 0.00366 | 0.00104 |
| 55-64 | **1.31326** | 0.00311 | 0.00641 | 0.02454 | 0.00141 | 0.00034 | 0.00362 | 0.90373 | 0.22477 | 0.00199 | 0.00014 | 0.10437 | 0.03505 | 0.00189 | 0.00189 |
| 65-74 | **1.75392** | 0.01415 | 0.01044 | 0.01932 | 0.00048 | 0.00026 | 0.00442 | 0.93449 | 0.49312 | 0.00199 | -0.00048 | 0.26647 | 0.01099 | -0.00222 | 0.00049 |
| 75+ | **1.41589** | 0.04282 | 0.01652 | 0.00537 | 0.00150 | -0.01998 | 0.00504 | 0.44456 | 0.49277 | 0.00108 | -0.00065 | 0.43870 | 0.00420 | -0.01614 | 0.00011 |
| Total | **5.99000** | **0.05791** | **0.05025** | **0.07692** | **0.16395** | **-0.01959** | **0.01825** | **3.01394** | **1.08080** | **0.01989** | **0.00009** | **0.87836** | **0.61875** | **-0.01263** | **0.04314** |

Source: Author computations using data from the Human Mortality Database and from the Office for National Statistics (nd b), The 20th Century Mortality Files, 1901-2000 release. This is available at: [https://webarchive.nationalarchives.gov.uk/20150908090558/http://www.ons.gov.uk/ons/publications/re-reference-tables.html?edition=tcm%3A77-215593](https://webarchive.nationalarchives.gov.uk/20150908090558/http:/www.ons.gov.uk/ons/publications/re-reference-tables.html?edition=tcm%3A77-215593)

Note: Negative numbers mean that mortality is lower in that age group for males than females, and thus acts to offset the female advantage at other age groups.

Table C.12: Age-cause-contribution in years to the total sex gap in 1991 in England and Wales

| **1991 RESULTS** | | | | | | | | | | | | | | | |
| --- | --- | --- | --- | --- | --- | --- | --- | --- | --- | --- | --- | --- | --- | --- | --- |
| Age class | Total contribution | Genitourinary | Nervous system | Digestive | Perinatal &  congenital | Old age | Tuberculosis | Circulatory | Neoplasms | Infectious | Diarrhoea | Respiratory | External  causes | Other + Childbirth | Ill  defined |
| <1 | **0.13961** | -0.00004 | 0.00064 | 0.00220 | 0.07792 | 0.00000 | 0.00000 | 0.00129 | 0.00053 | 0.00166 | 0.00036 | 0.00602 | 0.00131 | 0.00204 | 0.04568 |
| 1-14 | **0.05734** | -0.00024 | -0.00215 | 0.00238 | 0.00512 | 0.00000 | 0.00019 | -0.00002 | 0.00907 | 0.00217 | -0.00041 | 0.00511 | 0.03441 | 0.00211 | -0.00040 |
| 15-24 | **0.28524** | -0.00003 | 0.01130 | 0.00022 | 0.00599 | 0.00000 | -0.00017 | 0.00470 | 0.01260 | 0.00185 | 0.00031 | 0.00604 | 0.22661 | 0.01507 | 0.00073 |
| 25-34 | **0.23017** | -0.00093 | 0.00696 | 0.00413 | -0.00091 | 0.00000 | 0.00000 | 0.01941 | -0.01000 | 0.00341 | -0.00035 | 0.00847 | 0.17871 | 0.02010 | 0.00118 |
| 35-44 | **0.24904** | -0.00050 | 0.00771 | 0.01621 | 0.00243 | -0.00020 | 0.00081 | 0.11433 | -0.05975 | 0.00415 | 0.00010 | 0.01134 | 0.12380 | 0.02659 | 0.00202 |
| 45-54 | **0.45542** | 0.00101 | 0.00540 | 0.02017 | 0.00248 | -0.00056 | 0.00148 | 0.36568 | -0.04786 | 0.00193 | -0.00009 | 0.01555 | 0.07764 | 0.01038 | 0.00221 |
| 55-64 | **0.97260** | 0.00227 | 0.00274 | 0.02059 | 0.00026 | 0.00060 | 0.00122 | 0.67446 | 0.17293 | 0.00143 | 0.00014 | 0.05097 | 0.03416 | 0.00914 | 0.00168 |
| 65-74 | **1.53811** | 0.00493 | 0.01693 | 0.02227 | 0.00029 | 0.00178 | 0.00249 | 0.84059 | 0.46512 | 0.00199 | 0.00024 | 0.15422 | 0.01723 | 0.00880 | 0.00125 |
| 75+ | **1.57247** | 0.03751 | 0.04872 | 0.00034 | 0.00131 | -0.07784 | 0.00464 | 0.54281 | 0.69679 | 0.00089 | -0.00033 | 0.34680 | 0.00636 | -0.03579 | 0.00026 |
| Total | **5.50000** | **0.04399** | **0.09826** | **0.08851** | **0.09490** | **-0.07621** | **0.01066** | **2.56324** | **1.23942** | **0.01948** | **-0.00004** | **0.60451** | **0.70023** | **0.05845** | **0.05462** |

Source: Author computations using data from the Human Mortality Database and from the Office for National Statistics (nd b), The 20th Century Mortality Files, 1901-2000 release. This is available at: [https://webarchive.nationalarchives.gov.uk/20150908090558/http://www.ons.gov.uk/ons/publications/re-reference-tables.html?edition=tcm%3A77-215593](https://webarchive.nationalarchives.gov.uk/20150908090558/http:/www.ons.gov.uk/ons/publications/re-reference-tables.html?edition=tcm%3A77-215593).

Note: Negative numbers mean that mortality is lower in that age group for males than females, and thus acts to offset the female advantage at other age groups.

Table C.13: Age-cause-contribution in years to the total sex gap in 2001 in England and Wales

| **2001 RESULTS** | | | | | | | | | | | | | | | |
| --- | --- | --- | --- | --- | --- | --- | --- | --- | --- | --- | --- | --- | --- | --- | --- |
| Age class | Total contribution | Genitourinary | Nervous system | Digestive | Perinatal &  congenital | Old age | Tuberculosis | Circulatory | Neoplasms | Infectious | Diarrhoea | Respiratory | External  causes | Other + Childbirth | Ill  defined |
| <1 | **0.08485** | -0.00090 | 0.00240 | -0.00051 | 0.05877 | 0.00000 | 0.00000 | 0.00204 | -0.00011 | 0.00656 |  | 0.00581 | 0.00124 | -0.00111 | 0.01066 |
| 1-14 | **0.01985** | -0.00002 | 0.00397 | -0.00036 | 0.00232 | 0.00000 | -0.00012 | -0.00048 | 0.00037 | 0.00179 |  | -0.00212 | 0.01390 | -0.00027 | 0.00088 |
| 15-24 | **0.22873** | -0.00004 | 0.00883 | 0.00148 | 0.00488 | 0.00000 | 0.00034 | 0.00649 | 0.00935 | -0.00065 |  | 0.00002 | 0.17556 | 0.01924 | 0.00322 |
| 25-34 | **0.26486** | -0.00011 | 0.00619 | 0.00655 | 0.00005 | 0.00000 | 0.00037 | 0.02240 | -0.00918 | 0.00236 |  | 0.00431 | 0.18772 | 0.03774 | 0.00647 |
| 35-44 | **0.22572** | -0.00109 | 0.00716 | 0.03111 | 0.00177 | 0.00010 | 0.00124 | 0.06971 | -0.04549 | 0.00682 |  | 0.00856 | 0.11811 | 0.02246 | 0.00526 |
| 45-54 | **0.37330** | 0.00050 | 0.00279 | 0.04903 | -0.00121 | 0.00041 | 0.00066 | 0.23784 | -0.03173 | 0.00491 |  | 0.01332 | 0.07461 | 0.01585 | 0.00633 |
| 55-64 | **0.73173** | -0.00025 | 0.00218 | 0.04208 | 0.00100 | 0.00048 | -0.00011 | 0.44151 | 0.14582 | 0.00261 |  | 0.04252 | 0.04066 | 0.01116 | 0.00208 |
| 65-74 | **1.17472** | 0.00531 | 0.01198 | 0.02201 | -0.00023 | 0.00458 | 0.00045 | 0.62305 | 0.36543 | 0.00293 |  | 0.10392 | 0.02074 | 0.01214 | 0.00242 |
| 75+ | **1.54624** | 0.02536 | 0.04318 | -0.01402 | 0.00004 | -0.32531 | 0.00421 | 0.60036 | 0.92384 | 0.00433 |  | 0.33653 | 0.00470 | -0.05448 | -0.00249 |
| Total | **4.65000** | **0.02876** | **0.08868** | **0.13736** | **0.06739** | **-0.31975** | **0.00704** | **2.00292** | **1.35829** | **0.03166** |  | **0.51287** | **0.63723** | **0.06271** | **0.03483** |

| Source: Author computations using data from the Human Mortality Database and from the Office for National Statistics (2017), The 21st Century Mortality Files. This is available at:<https://www.ons.gov.uk/file?uri=/peoplepopulationandcommunity/birthsdeathsandmarriages/deaths/datasets/the21stcenturymortalityfilesdeathsdataset/current/regdeaths2001to2016.xls>. |
| --- |

Notes: Negative numbers mean that mortality is lower in that age group for males than females, and thus acts to offset the female advantage at other age groups. Because of the modest contribution of the group Diarrhoea as a cause of death in 2001, it has been redistributed between Infectious diseases and Digestive diseases, depending on the specific cause.

Table C.14: Age-cause-contribution in years to the total sex gap in 2011 in England and Wales

| **2011 RESULTS** | | | | | | | | | | | | | | | | | |
| --- | --- | --- | --- | --- | --- | --- | --- | --- | --- | --- | --- | --- | --- | --- | --- | --- | --- |
| Age class | | Total contribution | Genitourinary | Nervous system | Digestive | Perinatal &  congenital | Old age | Tuberculosis | Circulatory | Neoplasms | Infectious | Diarrhoea | Respiratory | External  causes | Other + Childbirth | Ill  defined | |
| <1 | | **0.09657** | -0.00065 | 0.00198 | 0.00035 | 0.07549 | 0.00000 | 0.00000 | 0.00338 | -0.00155 | 0.00282 |  | 0.00529 | -0.00059 | -0.00167 | 0.01172 | |
| 1-14 | | **0.02586** | -0.00066 | 0.00086 | 0.00222 | -0.00043 | 0.00000 | -0.00022 | 0.00269 | 0.00994 | 0.00319 |  | 0.00012 | 0.00926 | -0.00182 | 0.00071 | |
| 15-24 | | **0.13008** | -0.00050 | 0.00625 | 0.00105 | 0.00150 | 0.00000 | 0.00031 | 0.00669 | 0.00553 | 0.00011 |  | -0.00146 | 0.10889 | -0.00145 | 0.00317 | |
| 25-34 | | **0.17713** | -0.00043 | 0.00737 | 0.00551 | 0.00159 | 0.00000 | 0.00000 | 0.01977 | -0.01242 | 0.00102 |  | 0.00276 | 0.14386 | 0.00205 | 0.00606 | |
| 35-44 | | **0.24449** | -0.00089 | 0.00836 | 0.03492 | -0.00190 | 0.00000 | 0.00032 | 0.06906 | -0.03688 | 0.00343 |  | 0.00707 | 0.14717 | 0.00541 | 0.00844 | |
| 45-54 | | **0.31133** | -0.00040 | 0.00797 | 0.05687 | 0.00079 | -0.00021 | 0.00144 | 0.16160 | -0.04483 | 0.00484 |  | 0.01339 | 0.09226 | 0.01196 | 0.00567 | |
| 55-64 | | **0.59127** | 0.00257 | 0.00197 | 0.05809 | 0.00175 | 0.00119 | 0.00096 | 0.30868 | 0.11229 | 0.00484 |  | 0.03883 | 0.04440 | 0.01011 | 0.00558 | |
| 65-74 | | **0.89575** | 0.00091 | 0.01543 | 0.03370 | -0.00033 | 0.00569 | 0.00036 | 0.37765 | 0.33605 | 0.00273 |  | 0.08857 | 0.02593 | 0.00557 | 0.00349 | |
| 75+ | | **1.41752** | -0.00422 | 0.02511 | -0.04745 | 0.00099 | -0.76359 | 0.00411 | 0.48628 | 1.41681 | -0.02045 |  | 0.37747 | 0.02677 | -0.08232 | -0.00198 | |
| Total | | **3.89000** | **-0.00428** | **0.07530** | **0.14526** | **0.07945** | **-0.75693** | **0.00728** | **1.43580** | **1.78494** | **0.00253** |  | **0.53202** | **0.59795** | **-0.05217** | **0.04285** | |
| Source: Author computations using data from the Human Mortality Database and from the Office for National Statistics (nd a), Deaths registered in England and Wales, 2011 - Table 5. This is available at: [www.ons.gov.uk/ons/rel/vsob1/mortality-statistics--deaths-registered-in-england-and-wales--series-dr-/2011/dr-tables-2011.xls](http://www.ons.gov.uk/ons/rel/vsob1/mortality-statistics--deaths-registered-in-england-and-wales--series-dr-/2011/dr-tables-2011.xls). | | | | | | | | | | | | | | | |  |  |

Notes: Negative numbers mean that mortality is lower in that age group for males than females, and thus acts to offset the female advantage at other age groups. Because of the modest contribution of the group Diarrhoea as a cause of death in 2011, it has been redistributed between Infectious diseases and Digestive diseases, depending on the specific cause.
